# Comparative analysis identifies genetic and molecular factors associated with prognostic clusters of PANoptosis in glioma, kidney and melanoma cancer

**DOI:** 10.1038/s41598-023-48098-1

**Published:** 2023-11-28

**Authors:** Raghvendra Mall, Thirumala-Devi Kanneganti

**Affiliations:** 1 Department of Immunology, St. Jude Children’s Research Hospital, MS #351, 262 Danny Thomas Place, Memphis, TN 38105-2794 USA; 2 Present Address: Biotechnology Research Center, Technology Innovation Institute, P.O. Box 9639, Abu Dhabi, United Arab Emirates

**Keywords:** Cancer, Computational biology and bioinformatics

## Abstract

The importance of inflammatory cell death, PANoptosis, in cancer is increasingly being recognized. PANoptosis can promote or inhibit tumorigenesis in context-dependent manners, and a computational approach leveraging transcriptomic profiling of genes involved in PANoptosis has shown that patients can be stratified into PANoptosis High and PANoptosis Low clusters that have significant differences in overall survival for low grade glioma (LGG), kidney renal cell carcinoma (KIRC) and skin cutaneous melanoma (SKCM). However, the molecular mechanisms that contribute to differential prognosis between PANoptosis clusters require further elucidation. Therefore, we performed a comprehensive comparison of genetic, genomic, tumor microenvironment, and pathway characteristics between the PANoptosis High and PANoptosis Low clusters to determine the relevance of each component in driving the differential associations with prognosis for LGG, KIRC and SKCM. Across these cancer types, we found that activation of the proliferation pathway was significantly different between PANoptosis High and Low clusters. In LGG and SKCM, we also found that aneuploidy and immune cell densities and activations contributed to differences in PANoptosis clusters. In individual cancers, we identified important roles for barrier gene pathway activation (in SKCM) and the somatic mutation profiles of driver oncogenes as well as hedgehog signaling pathway activation (in LGG). By identifying these genetic and molecular factors, we can possibly improve the prognosis for at risk-stratified patient populations based on the PANoptosis phenotype in LGG, KIRC and SKCM. This not only advances our mechanistic understanding of cancer but will allow for the selection of optimal treatment strategies.

## Introduction

One of the founding hallmarks of cancer is the ability to resist cell death^1,2^. Several regulated cell death (RCD) pathways have been identified^3^, with the three most well characterized RCD pathways being apoptosis, pyroptosis and necroptosis. Though these have generally been thought of as segregated pathways, extensive context-dependent crosstalk among RCD molecular components exists^4–8^, thereby creating a knowledge gap in our mechanistic understanding of RCD. Studies to address this gap led to the identification of PANoptosis^4–7,9–17^, a unique innate immune inflammatory RCD pathway that is driven by caspases and RIPKs and regulated by PANoptosome complexes. PANoptosis plays a key role in cancer and cancer therapies. For example, IRF1-dependent PANoptosis inhibits the development of colorectal cancer in murine models^18^, and cotreatment of TNF-α and IFN-γ induces PANoptotic cell death in human cancer cells^5^. Recently, a comprehensive transcriptomic analysis using publicly available data from The Cancer Genome Atlas (TCGA) enabled a better understanding of the prognostic implications of PANoptosis on overall survival (OS) and identified a PANoptosis gene set with therapeutically relevant markers^19^.

The PANoptosis gene set is defined as 27 genes including cytosolic sensors and adaptors (e.g., NLRs), effectors (e.g., caspases) and upstream regulators^19^, and tumor samples cluster into PANoptosis High and PANoptosis Low groups. Tumors in the PANoptosis High cluster highly express most of the 27 PANoptosis genes, which may indicate these tumors experience enhanced cell death; in contrast, tumors in the PANoptosis Low cluster have low average expression of PANoptosis genes. The PANoptosis phenotype is significantly associated with OS for low grade gliomas (LGG), kidney renal cell carcinoma (KIRC) and skin cutaneous melanoma (SKCM). Specifically, the PANoptosis High cluster is associated with significantly reduced OS in LGG and KIRC (highest hazards ratio and P-value << 0.001) and significantly improved OS in SKCM (lowest hazards ratio and P-value << 0.001)^19^. While transcriptomic profiling was used to differentiate the PANoptosis clusters^19^, the role of genetic, genomic, tumor microenvironment and molecular pathway functional characteristics in differentiating the PANoptosis clusters for these cancers (LGG, KIRC and SKCM), where PANoptosis phenotype was prognostically the most relevant, has not yet been elucidated.

To address this gap, we performed comparative analysis of distinct genetic and molecular factors between PANoptosis High and Low clusters for each of these cancers. Our goal was to elucidate the biological traits that drive the difference between the PANoptosis clusters in LGG, KIRC and SKCM cancers. Analyses included comparing somatic mutations in driver oncogenes^20^, assessing non-silent mutation rate and neoantigen load^21^, analyzing aneuploidy and microsatellite instability scores^22^ and performing deconvolution of bulk RNA-Seq data to identify different cell type compositions. We also contrasted the activation profiles of oncogenic pathways^23^ between the PANoptosis clusters. The PANoptosis scores were then correlated with pathway activations across cancer cell lines^24^ and in different cell types in a single cell transcriptomics dataset for a melanoma case study^25^. We observed that aneuploidy, differences in immune cell densities and activations as well as activation of oncogenic pathways were associated with the differences between PANoptosis clusters for LGG, KIRC and SKCM. Together, these results identify distinct biological characteristics which differentiate the PANoptosis clusters in LGG, KIRC and SKCM and advance our understanding of cancer disease mechanisms. Targeting or manipulating these genetic and molecular components may improve survival for patients with LGG and KIRC belonging to the PANoptosis High cluster, patients with SKCM belonging to the PANoptosis Low cluster, and could potentially help additional patient populations.

## Methods

### Data acquisition, filtering and normalization

RNA-Seq data from TCGA^26^ were downloaded and processed using TCGA biolinks (v2.22.3). The RNA-Seq data for low grade gliomas (LGG) and kidney renal cell carcinoma (KIRC) consisted of 516 and 533 solid primary tumor (TP) samples, respectively. Due to the lack of TP samples in skin cutaneous melanoma (SKCM), metastatic samples (TM) were included in the SKCM dataset allowing for a total of 469 tumor samples. Gene symbols were converted to the official HGNC gene symbols, and genes without gene symbols or gene information were excluded. This resulted in p = 18,268 genes for each cancer type. The samples were quantile normalized using preprocessCore (v1.56.0) and log_2_ transformed for further analysis. The processed and quality controlled RNAseq datasets for LGG, KIRC and SKCM were obtained from Mendeley^27^.

### Cancer cell lines

A total of 1,377 cancer cell lines along with their expression profiles were downloaded from DepMap portal (DepMap Public 21Q3). These cell lines belong to the Cancer Cell Line Encyclopedia^24^. The cell lines were filtered to include only those cell lines for which the primary disease associated was low grade glioma, renal cell carcinoma or melanoma, resulting in 35, 16 and 34 cancer cell lines, respectively. These cancer cell lines had inherent diversity in terms of the age, gender, cancer type (primary or metastasis) of the patients as well as their sample collection site.

### Single cell transcriptomics

Single cell transcriptomics datasets for the SKCM cancer type (SKCM scRNA-Seq) were downloaded from the GEO Accession viewer under accession ID GSE72056^25^. The SKCM scRNA-Seq consisted of single cells derived from six patients each with at least 50 malignant cells as well as their corresponding non-malignant (immune and endothelial) cells. The dataset consisted of a total of 3,700 cells. The Seurat (v4.1.1) package in R^28^ with default normalization steps was used to process the dataset. These steps include normalizing using the ‘LogNormalize’ method with a scale factor of 10,000 followed by selection of the top 3,000 genes with maximum variance using the ‘vst’ method and scaling the dataset. Principal Component Analysis (PCA)^29^ was then performed, with the number of principal components set to the default setting of 30. Then, the Unified Manifold Approximation and Projection (UMAP)^30^ method was run, resulting in the 2D coordinates for the single cells and allowing visualization of the SKCM dataset. The metadata for the cluster labels of the single cells were available and used for annotating the single cell clusters.

### PANoptosis clusters

An unsupervised consensus clustering based on a gene set of 27 PANoptosis genes^19^ was separately performed for each cancer type using the ConsensusClusterPlus (v.1.58.0) R package. This methodology has previously been shown to be successful in identifying optimal prognostic clusters for pancancer immunologic constant of rejection^23,31–34^, pancancer PANoptosis^19^ and gastric cancer pyroptosis-related signatures^35^. With the intent to compare cancer samples with a highly active PANoptosis phenotype against those having a relatively inactive PANoptosis phenotype, the cluster with the highest average expression of PANoptosis genes was designated as PANoptosis High, while the cluster with the lowest average expression of PANoptosis genes was designated PANoptosis Low; the remaining tumor samples were classified as PANoptosis Medium.

For LGG, the dataset consisted of 100 PANoptosis High and 145 PANoptosis Low tumor samples from TCGA. Similarly, for KIRC and SKCM cancers, the dataset comprised 270 and 146 PANoptosis High and 198 and 48 PANoptosis Low samples, respectively.

### PANoptosis score

Tumor samples (bulk RNA-Seq from TCGA and cancer cell lines from CCLE) were annotated with a PANoptosis score, defined as the single sample gene set enrichment score (ssGSEA) obtained from the GSVA (v1.42.0) R package^36^ using the ‘gsva’ function with the kernel density parameter set as ‘Gaussian’. To estimate the PANoptosis score for each cell in the single cell transcriptomics dataset, the ‘enrichIt’ function from escape (v1.6.0) package in R^37^ was used. The ‘enrichIt’ function implements ssGSEA specific to single cell transcriptomic data.

### Somatic mutation analysis

The somatic mutation analysis data^38^ were downloaded along with variant annotations from the Genomic Data Commons (GDC)^39^ portal. A list of 291 high-confidence driver genes was obtained^20^ which were frequently mutated in pancancer. The maftools (v2.12.0) R package^40^ was used, in particular the ‘subsetMaf’, ‘getGeneSummary’, ‘oncoplot’ and ‘plotmafSummary’ functions, to obtain and plot summary information comparing different types of somatic mutations in the driver oncogenes between the PANoptosis High and Low clusters for each cancer type. The aggregate somatic mutations in the driver oncogenes (top 20 most mutated genes) were compared between PANoptosis High and Low clusters using the Chi-square test^41^ to determine if the mutation profiles were independent in PANoptosis High and Low clusters for each of LGG, KIRC and SKCM cancers.

Moreover, mutation (non-silent mutation) rates and predicted neoantigen counts for TCGA patient samples were collected from a recent immunogenic analysis^21^. Mutation rate and single nucleotide variant (SNV) neoantigen counts were log_10_-transformed and their distribution across PANoptosis clusters was plotted using the ggplot2 (v3.3.6) R package using the ‘geom_boxplot’ function. Differences between the PANoptosis High and Low clusters were calculated using the non-parametric Wilcoxon rank-sum test^42^ with multiple-testing correction using Bonferroni method^43^. We used the Bonferroni correctedP-value < 0.05 to determine statistically significance.

### Aneuploidy and microsatellite instability

Aneuploidy scores for each of the three cancer types were calculated as previously described^22^. Briefly, each tumor was scored for the presence of aneuploid chromosome arms after accounting for tumor ploidy, where aneuploidy is a surrogate measure for genomic instability^31^. The precomputed aneuploidy scores for each cancer type were then compared with the PANoptosis scores via a linear model. PANoptosis and aneuploidy score associations were evaluated by a linear model in R using the ‘lm’ function independently for each cancer type. The significance of association as determined by the linear model, independent of the direction of association, was also calculated for each cancer.

For analysis of microsatellite instability (MSI), the MSIsensor model along with the scores for all TCGA samples^44^ were used. The differences between the PANoptosis High and Low clusters were calculated using the non-parametric Wilcoxon rank-sum test with Bonferroni corrected i.e. multiple testing adjusted P-value < 0.05 to determine statistically significance.

### Tumor microenvironment deconvolution

To deconvolve the tumor samples belonging to PANoptosis High and Low clusters obtained from TCGA bulk RNA-Seq samples, the immunedeconv (v2.1.0) R package^45^ was implemented using the ‘deconvolute’ function with the method set as ‘quantiseq’ and parameter ‘arrays’ set to FALSE, suggesting that it does not perform quantile normalization. Quantile-normalized non-log_2_ transformed TPM gene expression was provided as input to obtain the different cell type fractions for each tumor sample^46^.

### ESTIMATE analysis

ESTIMATE^47^ is tool for predicting tumor purity and the presence of infiltrating stroma and immune cells in tumor samples using gene expression data. The ESTIMATE algorithm also uses ssGSEA to generate three scores including stroma score (presence of stroma in tumor sample), immune score (infiltration of immune cells in tumor sample) and estimate score (tumor purity). Scores were downloaded from TCGA tumor samples^48^, and a Wilcoxon rank-sum test was performed to compare the distribution of each of these scores between the PANoptosis High and Low clusters for the cancers of interest. The data were plotted as a boxplot using ‘geom_boxplot’ function from ggplot2 package in R.

### Gene set enrichment analysis

To determine the enrichment of specific gene sets, either reflecting the immune cell types or specific oncogenic pathways, a single sample gene set enrichment analysis (ssGSEA) was performed using the ‘gsva’ method with kernel density function parameter set to Gaussian kernel. To estimate the PANoptosis pathway and other oncogenic pathway activity for each cell in the single cell transcriptomics dataset, the ‘enrichIt’ function from escape (v1.6.0) package in R^37^ was used.

Immune cell-specific signatures were used as gene sets^49^ using ssGSEA to estimate and compare immune cell activations between PANoptosis High and Low clusters for each of the three cancers of interest. Gene sets used to determine activation of specific tumor-related pathways were obtained from multiple sources. Initially 24 hallmark pathways^50^ were selected, as they were regularly dysfunctional (positively or negatively activated) in cancer. Subsequently, non-redundant pathways from Ingenuity Pathway Analysis (IPA) platform were added to this collection^31^, resulting in the inclusion of 21 IPA pathways. Finally, several pathways were included that have previously been hypothesized to associate with cancer immune phenotypes, including Hypoxia/Adenosine immune cell suppression, immunogenic cell death, NOS1 signature, PI3Kgamma signature and SHC1/pSTAT3 signatures^51^, barrier genes^52^, the proliferation gene signature^53^ and genes upregulated in MAPK-mutated breast cancer^54^, resulting in a total of 54 oncogenic pathways. Here, the term ‘activity’ is analogous to ‘enrichment’. The functional differences in terms of pathway activities between the PANoptosis High and Low clusters were compared for LGG, KIRC and SKCM cancers using Wilcoxon rank-sum test with Bonferroni corrected P-values highlighted as asterisks.

### Pathway activity and correlation matrix

The average and difference in activity across immune cell types and oncogenic pathways between PANoptosis High and Low clusters for LGG, KIRC and SKCM cancers were plotted using the ComplexHeatmap (v2.10.0) R package^55^. The correlation between the activity of oncogenic pathways and PANoptosis scores for the bulk RNA-Seq from TCGA, the cell lines from CCLE and single cell transcriptomics was calculated using the Pearson correlation and plotted using ComplexHeatmap. The significance of correlations was estimated using the ‘cor.test’ function with the method parameter set as Pearson correlation. The significance of difference in activity between PANoptosis High and Low clusters was estimated using Wilcoxon rank-sum test (‘wilcox.test’ function) and adjusted for multiple testing using the Bonferroni correction method.

### Flowchart and study reproducibility

A flow chart highlighting the study design including comparative analysis of distinct genetic, genomic, tumor microenvironment and pathway characteristics between the PANoptosis High versus Low clusters for each cancer is illustrated at Supp. Fig. 1.

All the scripts and data required to reproduce the analysis findings are available at: https://data.mendeley.com/datasets/7x237xf2m3/1 along with a README.txt file to replicate the results.

## Results

### Comparison of somatic mutations in genes between PANoptosis high and low clusters

**Figure 1:**
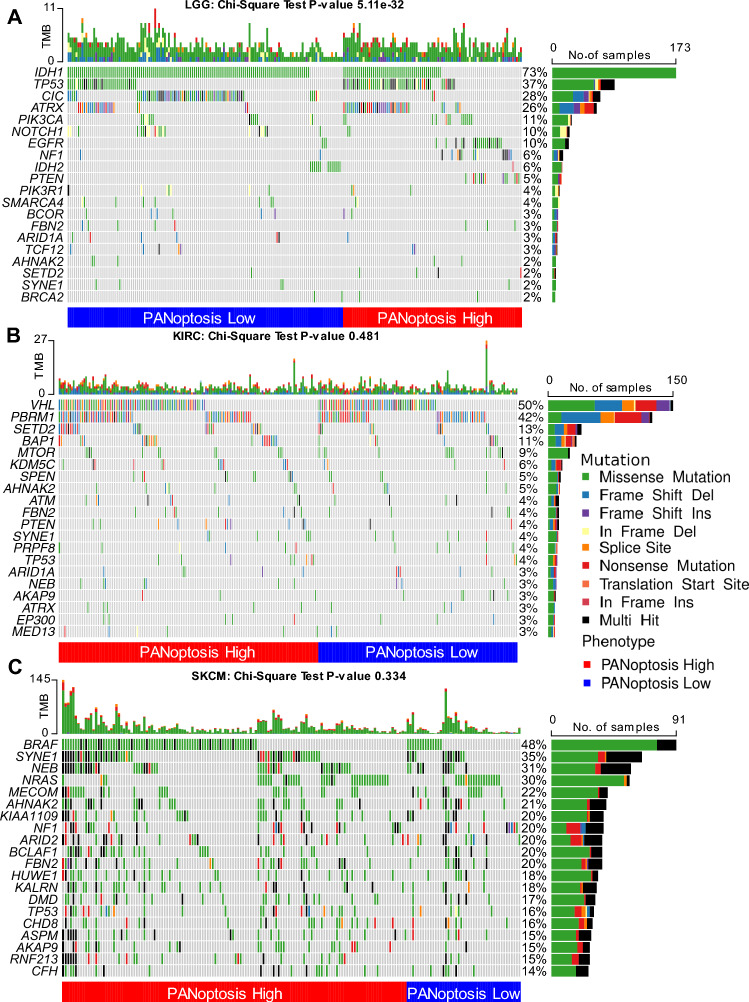
Comparison of somatic mutations in driver genes between PANoptosis High and PANoptosis Low clusters. (**A**–**C**) Total somatic mutations in the top 20 most mutated driver oncogenes compared using Chi-square test for LGG (**A**), KIRC (**B**) and SKCM (**C**) cancers. The class of somatic variants including Missense, Frame Shift Deletion (Frame Shift Del), Frame Shift Insertion (Frame Shift Ins), In Frame Deletion (In Frame Del), Splice Site, Nonsense Mutation, Translation Start Site, In Frame Insertion (In Frame Ins) and Multi Hit were color coded. Samples from the PANoptosis High group are over the red portion of the bar, and samples from the PANoptosis Low group are over the blue portion of the bar.

High and low expression of PANoptosis genes are associated with significant differences in OS for patients with LGG, KIRC and SKCM, but the molecular mechanisms driving these differences remain unclear. To determine whether somatic mutations in driver oncogenes contributed to the difference between the PANoptosis High and Low clusters for LGG, KIRC and SKCM cancers, we performed a comprehensive somatic mutation analysis. We first compared the total somatic mutations of high-confidence driver oncogenes, a set of 291 frequently mutated genes in cancer^20^, using a Chi-square test of independence between the PANoptosis High and Low clusters for LGG (Fig. 1A), KIRC (Fig. 1B) and SKCM (Fig. 1C). The null hypothesis for the Chi-square test was that there should be no difference in the distribution of somatic mutation profiles between PANoptosis High and Low clusters. We observed that LGG was the only cancer type where the null hypothesis was rejected (P-value = 5.11 × 10^–32^); the total somatic mutations in the driver oncogenes between PANoptosis High and Low clusters were significantly different in LGG (Fig. 1A). For both KIRC (P-value = 0.481, Fig. 1B) and SKCM (P-value = 0.334, Fig. 1C), the differences in somatic mutations in PANoptosis High and Low clusters were not significant. We also compared the somatic mutations in PANoptosis genes between the PANoptosis High and Low clusters for LGG (Supp. Fig. 2A), KIRC (Supp. Fig. 2B) and SKCM (Supp. Fig. 2C) cancers, but owing to small numbers of mutations in these genes, we could not perform the Chi-square test. These results suggested that mutations in PANoptosis genes could not significantly drive the differences between PANoptosis High and Low clusters.

Among the high-confidence driver oncogenes, the difference between the PANoptosis High and Low clusters for LGG was driven by *CIC*, *NOTCH1*, *EGFR*, *NF1*, *IDH2* and *PTEN* oncogenes (Fig. 1A and Supp. Table 1). *CIC*, *NOTCH1* and *IDH2* genes were frequently mutated in the PANoptosis Low cluster with a high number of frame shift deletions, missense and in-frame deletion variants (Supp. Table 1). *EGFR* and *NF1* genes were more frequently mutated in the PANoptosis High cluster, with a high number of missense mutations, frame shift deletions and nonsense mutations (Supp. Table 1). Moreover, the median number of nonsense mutations per tumor sample (as well as the total number) in the driver oncogenes was higher in the PANoptosis High cluster, where there was worse survival, versus the PANoptosis Low cluster for LGG; all other variants had similar frequency (Supp. Fig. 3).

For KIRC, we observed several missense mutations, frame shift deletions and nonsense mutations in both PANoptosis High and Low tumor samples (Supp. Fig. 4 and Supp. Table 2). The oncogenes *VHL*, *PBRM1*, *SETD2*, *BAP1* and *MTOR* were among the most frequently mutated oncogenes for both PANoptosis High and Low clusters (Supp. Fig. 4 and Supp. Table 2), thereby suggesting limited contribution of somatic mutations in driving the differences between PANoptosis High and PANoptosis Low clusters for KIRC.

Similarly, for the SKCM cancer type, we observed large numbers of missense, nonsense and splice site somatic mutations (Supp. Fig. 5 and Supp. Table 3). Additionally, oncogenes such as *BRAF*, *SYNE1*, *NEB*, *AHNAK2*, *NRAS* and *FBN2* were frequently mutated for both the PANoptosis High and PANoptosis Low clusters (Supp. Fig. 5 and Supp. Table 3), resulting in a P-value = 0.334 for the Chi-square test of independence (Fig. 1C). In comparison to LGG and KIRC cancers, a myriad of oncogenes was frequently mutated in SKCM (Supp. Table 3).

Taken together, these results highlight that somatic mutation profiles of driver oncogenes were significantly different and could contribute to the differences in PANoptosis High versus PANoptosis Low clusters for LGG, but not for KIRC or SKCM.

### Comparison of genomic alterations between PANoptosis high and low clusters

**Figure 2:**
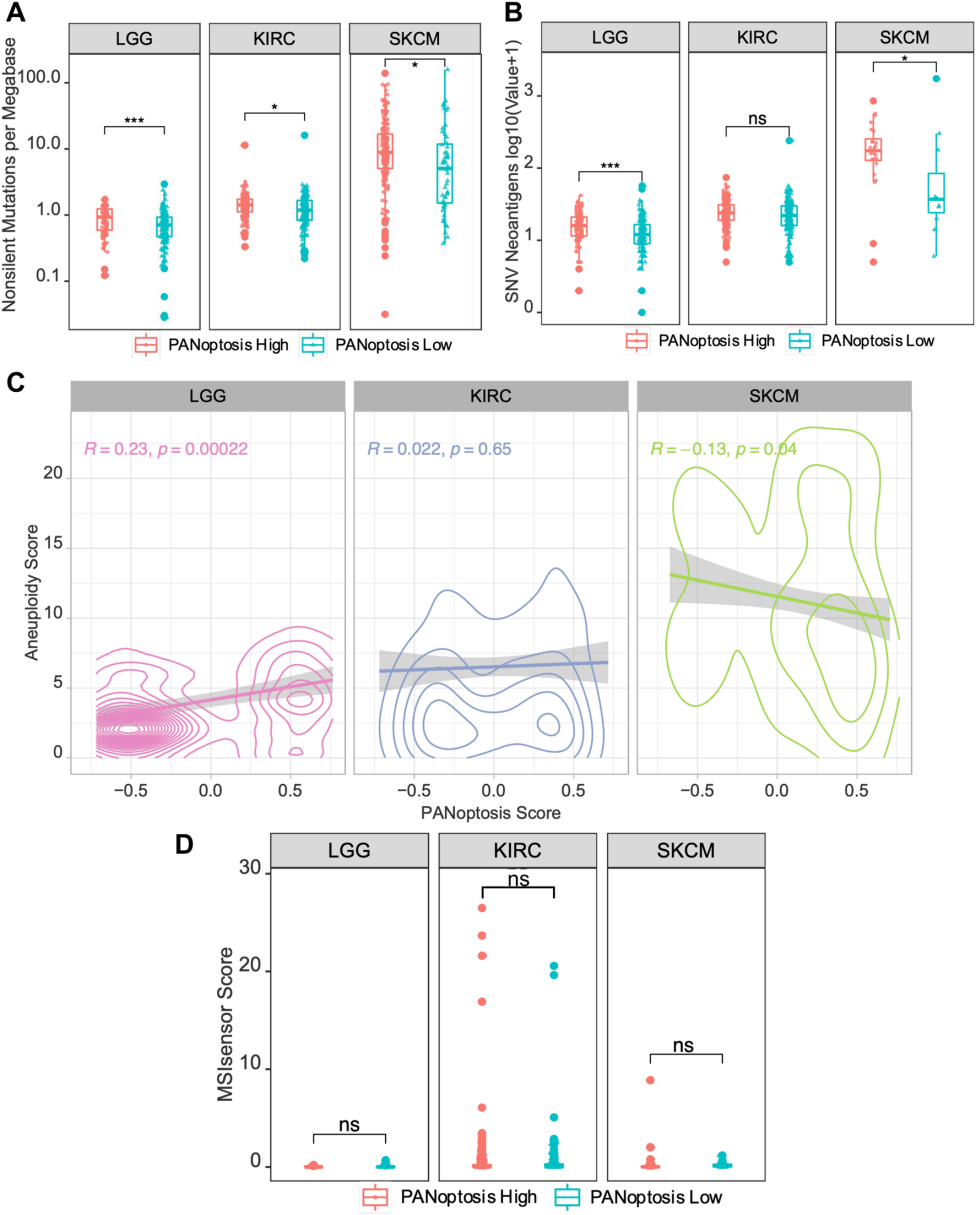
Comparison of genomic stability measures between PANoptosis High and PANoptosis Low clusters for LGG, KIRC and SKCM. (**A**) Boxplot of log transformed non-silent mutation rate per PANoptosis cluster for each cancer type of interest. The non-silent mutation rate for each tumor sample was highlighted along with the boxplot as jitters. (**B**) Boxplot of log transformed single nucleotide variation (SNV) neoantigen counts per PANoptosis cluster. The SNV neoantigen counts for each tumor sample were highlighted along with the boxplot as jitters. (**C**) Pearson correlation between aneuploidy score and PANoptosis score for each of the three cancer types. (**D**) Boxplot of MSIsensor score per PANoptosis cluster. The MSIsensor scores for each tumor sample were highlighted along with boxplot as jitters. For (**A**,**B**,**D**), the statistical significance reported was based on Bonferroni-corrected significance of difference estimated via a non-parametric Wilcoxon rank-sum test with *P-value ∈ (1e−2, 0.5*1e−2]; ***P-value < 1e−3; ns, not significant.

We next investigated the role of genomic alterations such as neoantigen count, aneuploidy and MSI in PANoptosis High and PANoptosis Low clusters. It has previously been shown that the mean neoantigen count, also referred to as the tumor mutation burden (TMB), for each cancer type strongly correlates with the mean mutation rate in TCGA^23,31^. While the mean non-silent mutation (NSM) rate was significantly higher in PANoptosis High tumors for LGG (∆μ(NSM) = 0.20), KIRC (∆μ(NSM) = 0.12) and SKCM (∆μ(NSM) = 1.98) cancers, the absolute difference in mutation rate was much smaller for LGG and KIRC in comparison to SKCM (Fig. 2A). Similarly, the TMB was significantly higher in PANoptosis High tumors for LGG (∆μ(TMB) = 0.11) and SKCM (∆μ(TMB) = 0.46) cancer types, where the absolute difference in TMB was much smaller for LGG compared to SKCM (Fig. 2B). However, these differences failed to explain the divergent association of PANoptosis with survival (PANoptosis High beneficial for SKCM and detrimental for LGG and KIRC), as the mutation rate and SNV neoantigen counts were consistently higher in PANoptosis High clusters for LGG, KIRC and SKCM cancer types.

Additionally, we analyzed the association between genomic instabilities, including aneuploidy and microsatellite instability (MSI), with respect to the PANoptosis groups. We compared the individual tumor aneuploidy score and the PANoptosis score across cancer samples for each cancer type. Aneuploidy scores^22^ had a significant positive association with the PANoptosis scores for LGG (R = 0.23, P-value = 0.00022) and KIRC (R = 0.022, P-value = 0.65) and a negative association for SKCM (R =  − 0.13, P-value = 0.04) (Fig. 2C). This suggests that there are more chromosomal instabilities in PANoptosis High tumor samples for LGG, and more chromosomal instabilities in PANoptosis Low tumor samples for SKCM. This aligned well with the divergent prognostic association of PANoptosis phenotype for LGG in comparison to SKCM.

We further analyzed MSI, a pattern of hypermutation that occurs at the genomic microsatellites or towards the end of chromosomes and is caused by defects in the mismatch repair system. Mismatch repair deficiency leads to high MSI, and has been shown to be beneficial for immunotherapy^56^. We compared the MSIsensor scores obtained from MSIsensor^44^ between the PANoptosis clusters for LGG, KIRC and SKCM cancers (Fig. 2D). We observed no significant difference in the MSIsensor scores between the PANoptosis High and Low groups for all three cancers.

Taken together, these results indicate that non-silent mutation rate and SNV neoantigen count may play a role in driving the difference between the PANoptosis clusters for SKCM, while aneuploidy could possibly play a role in the divergent association of PANoptosis with OS in LGG and SKCM cancers.

### Comparison of tumor microenvironment composition and activation between PANoptosis clusters

To further analyze the biological factors contributing to the differences between the PANoptosis High and Low groups in LGG, KIRC and SKCM, we compared the fraction of different cell type populations between the PANoptosis clusters through the quanTIseq method^57^. The tumor samples belonging to PANoptosis High and Low clusters obtained from TCGA were bulk RNA-Seq samples that require deconvolution to attain a better picture of the tumor microenvironment and its potential contribution to the differences between the PANoptosis High and Low clusters. While many deconvolution techniques exist^58–60^, quanTIseq was used here because it is specifically designed for RNA-Seq data, has high agreement with the cell fractions computed with flow cytometry for both immune and uncharacterized cells and allows for a comparison of immune cell type fractions across samples, thereby overcoming limitations encountered by other methods^45^.

**Figure 3:**
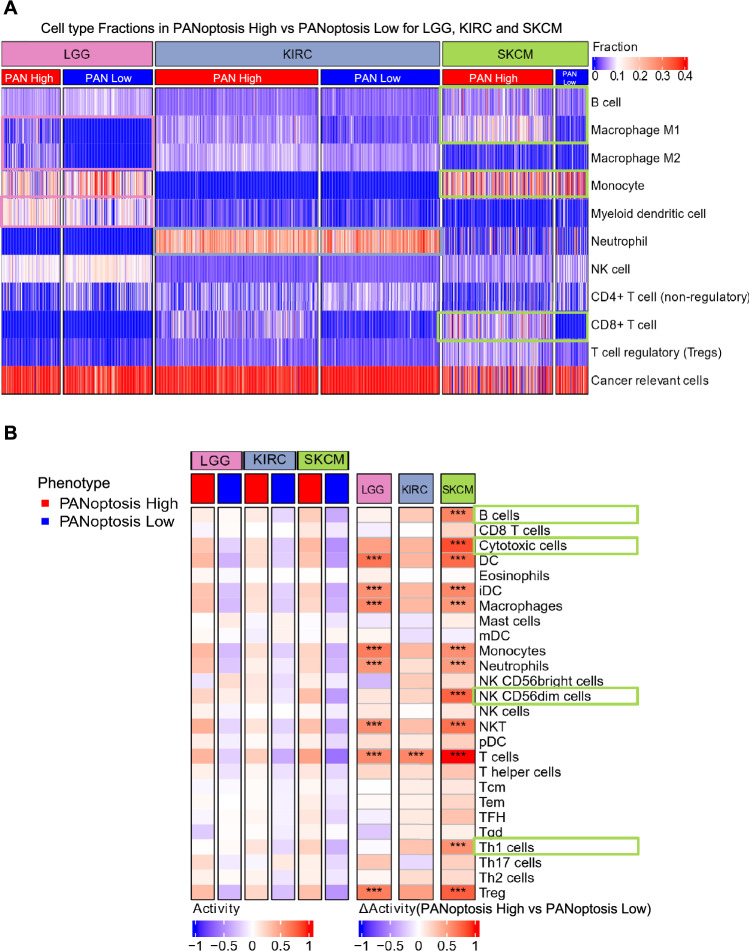
Comparison of cell type fractions and cell type activation between PANoptosis High versus PANoptosis Low groups for LGG, KIRC and SKCM. (**A**) Cell type fractions in PANoptosis High and PANoptosis Low clusters for LGG, KIRC and SKCM cancers estimated using quanTIseq deconvolution method on bulk RNA-Seq gene expression profiles. (**B**) Heatmap of enrichment (activation) values for cell type specific immune-signature using the ssGSEA method. The difference in activity profiles of cell types between PANoptosis High versus PANoptosis Low samples were highlighted as ∆Activity, and significance in difference was estimated using a Wilcoxon rank-sum test with ***P-value < 1e−3. Samples from the PANoptosis High group are depicted below the red bar and samples from the PANoptosis Low group are depicted below the blue bar.

Gene expression profiles and the signature matrix specific to 11 cell types^45^ were used to deconvolute the abundance of different immune cell types in tumor samples using the quanTIseq method^45^. We found that similar cell fractions (CF) of B cells, monocytes, myeloid dendritic cells, neutrophils, NK cells, CD4 + T cell (non-regulatory) and cancer-relevant cells were observed between PANoptosis High and Low clusters in LGG, KIRC and SKCM cancers, indicating that these cell populations are similar with respect to PANoptosis phenotype (Fig. 3A). However, increased M2 macrophages (∆μ(CF) = 0.04), commonly deemed as pro-tumorigenic macrophages^61^, and increased myeloid dendritic cells (∆μ = 0.046), frequently considered to promote tumorigenesis and immunosuppression^62^, were observed in PANoptosis High tumor samples compared to PANoptosis Low samples for LGG (Fig. 3A). Similarly, increased M1 macrophage (∆μ(CF) = 0.093), historically regarded as anti-tumorigenic^61^, as well as increased infiltrating cytotoxic CD8 + T cell (∆μ(CF) = 0.091) and B cell (∆μ(CF) = 0.026) fractions, and decreased monocyte (∆μ(CF) =  − 0.077) fractions, which are often immunosuppressive, were observed in the PANoptosis High group in comparison to PANoptosis Low cluster for SKCM (Fig. 3A). For KIRC, increased cytotoxic CD8 + T cell (∆μ(CF) = 0.051) fractions were observed but no significant difference existed in regulatory Tregs (∆μ(CF) = 0.01) or non-regulatory CD4 + T cell (∆μ(CF) = 0.01) fractions between PANoptosis High vs Low clusters (Fig. 3A). It has previously been shown that a higher population of exhausted/polarized CD8 + T cells was associated with higher grades of renal cell carcinoma and worse survival prognoses^63^.

Using gene expression signatures specific to 24 cell types^49^, we estimated the cell activations (CA) of different immune cell types using a ssGSEA method for each sample and compared the immune CA between PANoptosis High vs Low clusters for LGG, KIRC and SKCM. For LGG, KIRC and SKCM, we observed increased activation of cytotoxic T cells, dendritic cells (DC and iDC), macrophages, monocytes, neutrophils, NK CD56dim cells, NKT, pDC, T cells, T helper cells and T-regulatory (Treg) cells in the PANoptosis High samples compared to PANoptosis Low samples (Fig. 3B). However, in SKCM, there were significantly differentially activated B cells (∆μ(CA) = 0.62, Bonferonni corrected P-value = 2.8 × 10^−22^) and Th1 cells (∆μ(CA) = 0.56, Bonferroni corrected P-value = 1.69 × 10^−26^) as well as cytotoxic cells (∆μ(CA) = 0.85, Bonferroni corrected P-value = 1.25 × 10^−26^) and NK CD56dim cells (∆μ(CA) = 0.77, Bonferroni corrected P-value = 7.3 × 10^−24^), which suggests more overall anti-tumor immune cell infiltration in the PANoptosis High versus PANoptosis Low samples for SKCM (Fig. 3B).

Finally, we compared the differences in tumor purity (ESTIMATE method), immune and stroma scores between the PANoptosis High and PANoptosis Low clusters for LGG, KIRC and SKCM cancers to determine whether these scores could contribute to the differences between the two clusters. The ESTIMATE, immune and stroma scores were significantly higher in PANoptosis High samples when compared to PANoptosis Low samples for each of the three cancers, suggesting a more immune-infiltrated tumor and a microenvironment with tumors of higher purity for samples belonging to the PANoptosis High cluster (Supp. Fig. 6). However, these differences could not explain the divergent prognosis of PANoptosis with survival (PANoptosis High beneficial for SKCM and detrimental for LGG and KIRC).

Taken together, these results suggested that immune cell type density and activations in the tumor microenvironment contribute to the significant differences in the PANoptosis High versus PANoptosis Low clusters, specifically for LGG and SKCM.

### Comparison of activations of oncogenic pathways between PANoptosis High versus PANoptosis Low clusters

**Figure 4:**
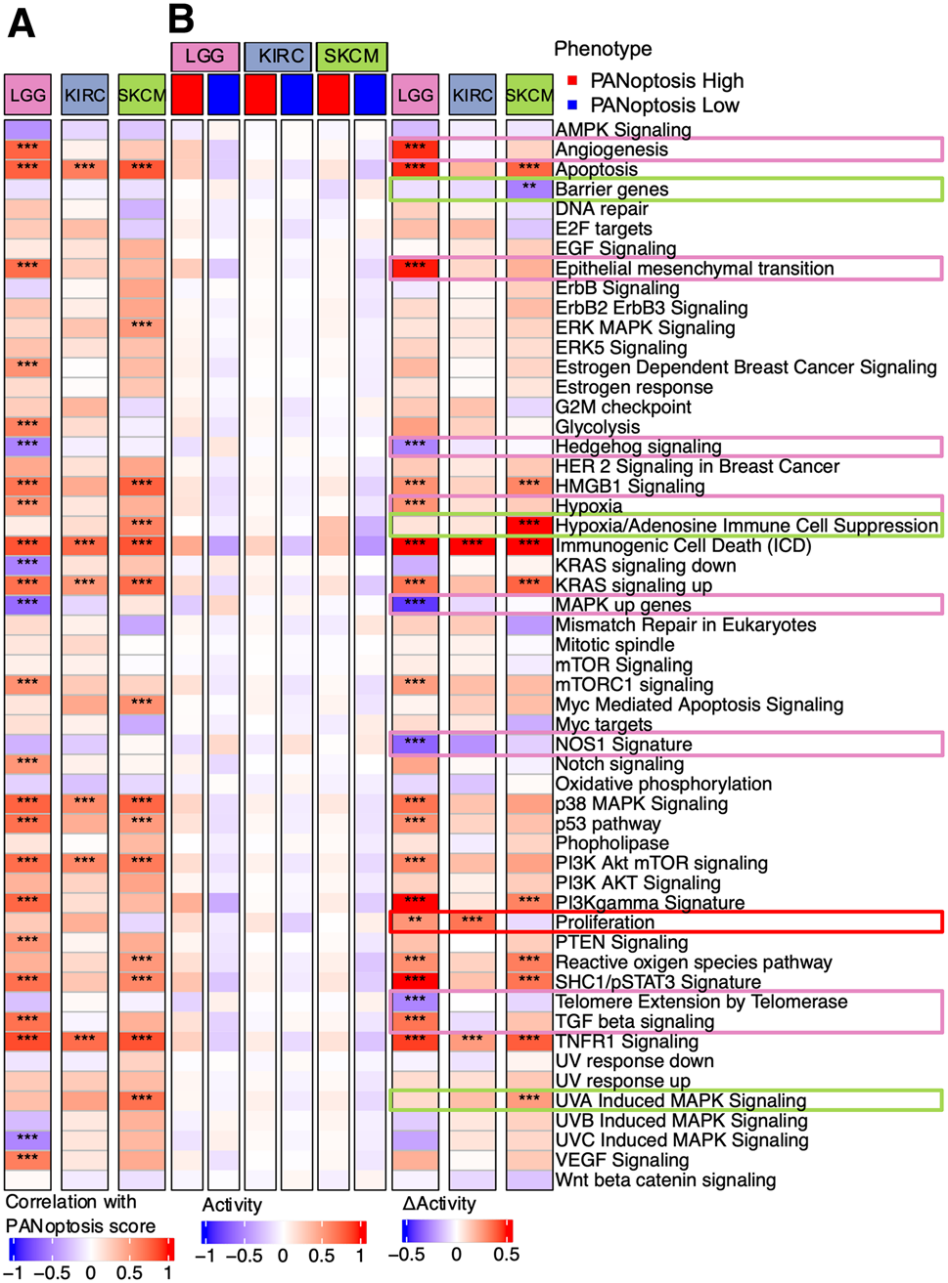
Comparison of oncogenic pathway activity between PANoptosis High versus PANoptosis Low tumors (bulk RNA-Seq) for LGG, KIRC and SKCM. (**A**) Pearson correlation coefficient between the PANoptosis activity and enrichment scores of the 54 oncogenic pathways^23^ for each of the three cancer types. The correlation co-efficient (R) was considered significant if |R|> 0.25 with ***P-value < 1e−5. (**B**) Heatmap of the average activation across bulk RNA-Seq tumor profiles for the oncogenic pathway estimated using ssGSEA for PANoptosis High and Low clusters for LGG, KIRC and SKCM cancers. The difference in average activity of oncogenic pathways between PANoptosis High versus PANoptosis Low groups was highlighted as ∆Activity and the significance in difference was estimated using a Wilcoxon rank-sum test with *P-value ∈ (1e−2, 0.5*1e−2]; **P-value ∈ (1e−3, 1e−2]; ***P-value < 1e−3. Samples from the PANoptosis High group are depicted below the red bar and samples from the PANoptosis Low group are depicted below the blue bar.

To examine the tumor intrinsic differences between PANoptosis High and PANoptosis Low clusters of LGG, KIRC and SKCM, we compared the enrichment of 54 oncogenic pathways between the groups. Pathway activations (PA) were estimated using the ssGSEA method on bulk RNA-Seq profiles (Supp. Fig. 7) and compared with the PANoptosis score for all tumor samples of each cancer type using the Pearson correlation (Fig. 4A). Moreover, we estimated the average and difference in activity (∆Activity) of the samples belonging to PANoptosis High and PANoptosis Low clusters, where the difference in activity was compared using Wilcoxon rank-sum test (Fig. 4B). Many pathways were differentially enriched between the two PANoptosis groups. For example, apoptosis, Immunogenic Cell Death (ICD), KRAS signaling up, p38 MAPK signaling, PI3K Akt mTOR signaling and TNFR1 signaling pathways had significant positive correlations with the PANoptosis score (R > 0.25, Bonferroni corrected P-value < 1 × 10^−5^, Fig. 4A) as well as a significant positive difference in enrichment between PANoptosis High and PANoptosis Low clusters for LGG, KIRC or SKCM individually (Fig. 4B). The apoptosis and ICD pathways had positive average activation in the PANoptosis High cluster and negative average activation in the PANoptosis Low cluster (Fig. 4B) for all three cancers; these pathways can be considered as validation of the PANoptosis phenotype since many of the genes in the PANoptosis gene set are also present in these pathways. However, these differences (positive in all three cancers) could not explain the divergent association of PANoptosis phenotype with survival.

We observed that there were several immunosuppressive pathways including angiogenesis (∆μ(PA) = 0.473, P-value = 3.35 × 10^−36^), epithelial mesenchymal transition (∆μ(PA) = 0.489, P-value = 7.43 × 10^−29^), hypoxia (∆μ(PA) = 0.275, P-value = 3.46 × 10^−27^) and TGF beta signaling (∆μ(PA) = 0.345, P-value = 3.05 × 10^−28^) which were distinctly activated (or positively correlated with PANoptosis score) in the PANoptosis High cluster as compared to the PANoptosis Low cluster for LGG (Fig. 4A,B). Similarly, several immunoregulatory pathways such as MAPK up genes (∆μ(PA) =  − 0.429, P-value = 2.33 × 10^−25^), NOS1 signature (∆μ(PA) =  − 0.338, P-value = 5.94 × 10^−8^), and telomere extension by telomerase (∆μ(PA) =  − 264, P-value = 5.12 × 10^−8^) were distinctly activated (or negatively correlated with PANoptosis score) in the PANoptosis Low cluster when compared to the PANoptosis High group for LGG (Fig. 4A,B). Furthermore, barrier genes (∆μ(PA) =  − 0.274, P-value = 3.34 × 10^−4^), UVA-induced MAPK signaling (∆μ(PA) = 0.255, P-value = 2.40 × 10^−17^) and hypoxia/adenosine immune cell suppression (∆μ(PA) = 0.65, P-value = 2.24 × 10^−15^) pathways were differentially enriched between the PANoptosis High and PANoptosis Low groups for SKCM cancer. Finally, the proliferation pathway was significantly differentially (positively) activated for LGG (∆μ(PA) = 0.271, P-value = 2.26 × 10^−4^) and KIRC (∆μ(PA) = 0.345, P-value = 9.02 × 10^−12^) and was also negatively activated for SKCM (∆μ(PA) =  − 0.08), thereby aligning with the divergent prognostic association of PANoptosis (PANoptosis High was detrimental for LGG and KIRC and beneficial for SKCM) for the three cancers of interest (Fig. 4B). Proliferation plays a prognostic role in cancer independent of the immune contexture^23,31^, and we observed that tumor samples with high average proliferation activity correlated with worse survival (Supp. Fig. 8).

**Figure 5:**
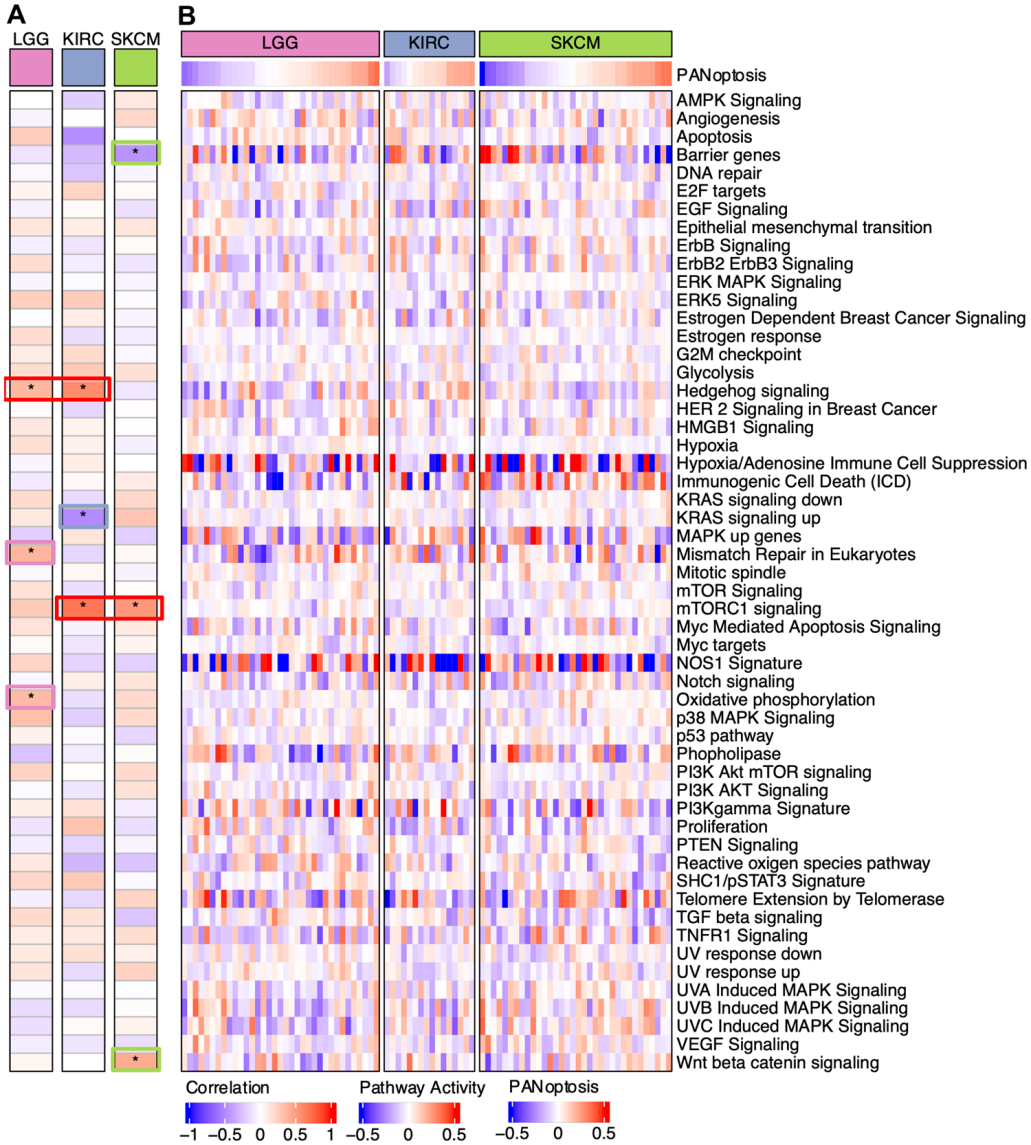
Comparison of oncogenic pathway activity and its correlation with PANoptosis scores of cancer cell lines for LGG, KIRC and SKCM. (**A**) Pearson correlation coefficient between the PANoptosis score and activity scores of the 54 oncogenic pathways^23^ across the cancer cell lines from CCLE for each of the three cancer types. The correlation co-efficient (R) was considered significant if |R|> 0.25 with *P-value ∈ (1e−3, 0.5*1e−2]. (**B**) For each cancer cell line, the PANoptosis score was estimated using the ssGSEA method. Heatmap of the pathway activities determined using ssGSEA across the cancer cell lines for LGG, KIRC and SKCM cancers is shown.

In addition to primary tumors, mechanistic information about cancer disease processes is often studied in cancer cell lines. While these cell lines have been the cornerstone of cancer research in experimental settings (in vitro systems), they have distinct genetic, genomic and functional properties compared to the primary tumors from which they were derived. This can make it difficult to translate in vitro findings into preclinical models^64–66^. There is lack of immune and stromal cells in established cell lines when compared to patient profiles, and what is observed in patient profiles might not always correlate with the observations from cell lines. Thus, we aimed to determine if any of the pathway activations (PA) and differences that we observed in primary tumor profiles for LGG, KIRC and SKCM aligned with the cancer cell lines obtained from CCLE (Fig. 5). We estimated the PANoptosis score and correlated it with the PA for all cancer cell lines belonging to glioma, kidney and melanoma lineages (Fig. 5A). The PA for each cell line was determined using the ssGSEA method on the gene expression profile associated with the cell line (Fig. 5B). We observed that the barrier genes pathway was significantly negatively correlated (R =  − 0.467, P-value = 0.006) with the PANoptosis score for melanoma, an observation consistent with primary tumor samples (Fig. 4B) and unique to melanoma (SKCM) cancer. Similarly, the mTORC1 signaling pathway (Fig. 5A) was positively correlated with the PANoptosis score for the cell lines belonging to each of the three cancers of interest, aligning with the observation from TCGA bulk RNA-Seq data (Fig. 4A). However, there were certain pathways such as hedgehog signaling (R = 0.371, P-value = 0.03 for LGG and R = 0.588, P-value = 0.02 for KIRC), oxidative phosphorylation (R = 0.353, P-value = 0.04 for LGG), Wnt beta catenin (R = 0.434, P-value = 0.01) and KRAS signaling up (R = -0.503, P-value = 0.05) which were specifically significantly correlated with the PANoptosis score across cancer cell lines (Fig. 5A) with contrasting correlation patterns to PANoptosis phenotype when compared to corresponding patient-derived tumors from TCGA (Fig. 4A).

Taken together, these results suggest that there are specific pathways whose activations could explain the differences between the PANoptosis clusters specifically for LGG and SKCM cancers. In particular, the proliferation pathway from bulk RNA-Seq and hedgehog signaling pathway in cancer cell lines aligned with the difference in survival association of PANoptosis phenotype for LGG and KIRC (PANoptosis High had worse survival) in comparison to SKCM (PANoptosis High had better survival).

### Comparison of activations of oncogenic pathways in melanoma single cell transcriptomics

**Figure 6:**
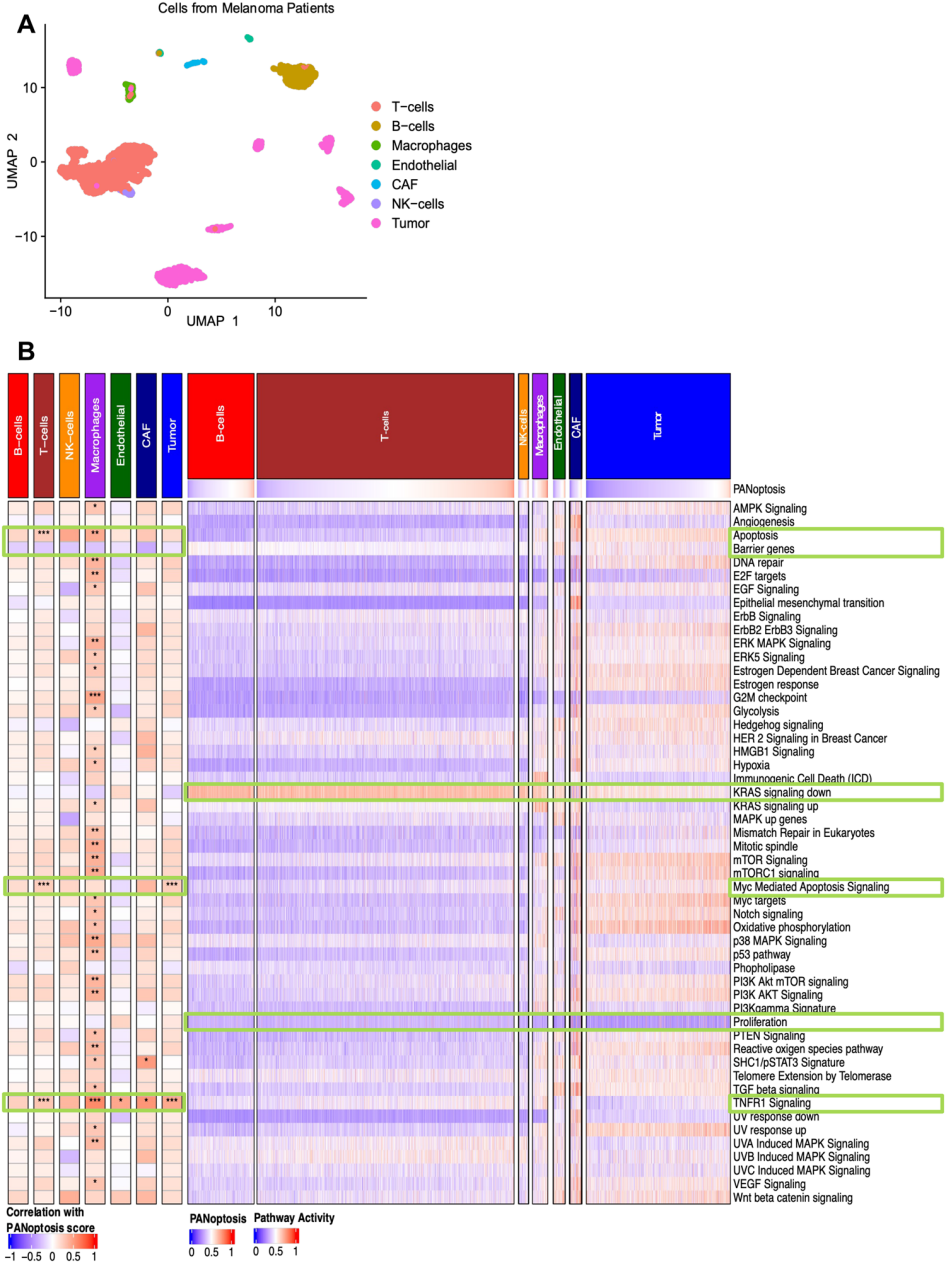
Single-cell analysis reveals oncogenic pathways correlated with PANoptosis score across immune, stromal and cancer cells in melanoma dataset. (**A**) UMAP plot highlighting the different cell type composition of the melanoma single cell transcriptomic dataset. (**B**) Pearson correlation between PANoptosis score and activity scores of the 54 oncogenic pathways across the immune, stroma and cancer cell types in the scRNA-Seq dataset. The correlation coefficients (R) were considered significant if |R|> 0.25 with *P-value ∈ (1e−3, 0.5*1e−2]; **P-value ∈ (1e−5, 1e−3]; ***P-value < 1e−5. Heatmap of the pathway activities determined using ‘enrichIt’ function for the different immune, cancer and stroma cell types and its comparison with the PANoptosis scores is shown.

Since, we studied the pathway activations for bulk RNA-seq and cancer cell lines, our next goal was to see how the pathway enrichments get impacted across different cell types in the tumor microenvironment. Hence, we investigated the enrichment of oncogenic pathways at the single cell resolution by analyzing a publicly available scRNA-Seq dataset for melanoma (GSE72056^25^) as a case study. The dataset was comprised of six heterogenous patients as highlighted by the segregated tumor samples for individual patients in the UMAP plot (Fig. 6A). However, the various immune cell type including T-cells, B-cells, macrophages and NK-cells for the six patients clustered together (Fig. 6A). Similarly, stroma-associated endothelial cells and cancer-associated fibroblasts (CAF) tended to cluster together in the UMAP plot (Fig. 6A).

PA was then compared across the different cell types with the PANoptosis score for each cell (Fig. 6B). Pathways such as apoptosis, Myc-mediated apoptosis signaling, SHC1/pSTAT3 signature and TNFR1 signaling were significantly positively correlated with the PANoptosis score across multiple cell types (Fig. 6B) and aligned well with the PA observed in primary tumor clusters via bulk RNA-Seq in SKCM cancer (Fig. 4B). Additionally, the barrier genes pathway was negatively correlated with PANoptosis score across all cell types, suggesting that cells with a higher PANoptosis score have lower barrier gene PA. This observation was also supported by the significant negative difference in average pathway activity between PANoptosis High and PANoptosis Low clusters for SKCM cancer (Fig. 4B) and significant negative correlation (with PANoptosis score) in melanoma cancer cell lines from CCLE (Fig. 5A). A lower activation score for the barrier gene pathway could suggest fewer barriers to immune infiltration in metastatic tumor profiles, thereby enhancing survival prognosis^67^. Moreover, the proliferation pathway was negatively enriched and the KRAS signaling down pathway was positively enriched across the majority of cells independent of the status of PANoptosis score (Fig. 6B), thereby providing insights into dysregulation of these pathways in patients with metastatic melanoma for individual cells at the single cell resolution.

Taken together, these results suggest that the oncogenic PA observed here at single cell resolution generally aligns with the pathway enrichments observed from bulk RNA-Seq for melanoma. Pathways relevant to cell death, such as apoptosis and Myc-mediated apoptosis signaling, were positively correlated with PANoptosis scores at both bulk (Fig. 4A) and single-cell levels across multiple cell types (Fig. 6B). Additionally, the barrier gene pathway could be a potential pathway that can be targeted in patients with low PANoptosis scores to drive survival benefit, although this requires further investigation.

## Discussion

A better understanding of the host-tumor relationship is imperative for developing effective therapeutic techniques and accurate stratification systems^70^, as it allows for a better understanding of the implications for at risk patient populations. In this work, we identified several contributing factors which could explain the differences between the PANoptosis clusters for LGG, KIRC and SKCM cancers. The PANoptosis High cluster had signficantly better survival for SKCM cancer and signficantly worse survival for LGG and KIRC cancers when compared to PANoptosis Low cluster for each of these three cancers.

In all three tumor types, the mutational load or TMB was usually higher in the PANoptosis High groups. In hypermutated tumors (SKCM—PANoptosis High cluster), abundant neoantigens are likely to facilitate immune recognition^71^. However, neither the mutational load or TMB could align with the differential prognosis of PANoptosis clusters between LGG, KIRC and SKCM cancers. In contrast, the measure of genomic instability, the aneuploidy score^22^, did align with the differential prognosis of PANoptosis clusters for LGG and SKCM cancers. A majority of human solid tumors are aneuploid^72^, and our findings suggest that more tumors in the PANoptosis High cluster for LGG and PANoptosis Low cluster for SKCM were aneuploid (Fig. 2C). Moreover, in^19^, it was shown that the PANoptosis High cluster had a worse survival prognosis for LGG cancer, while the PANoptosis Low cluster had a worse survival prognosis for SKCM cancer. Thus, higher aneuploidy scores are associated with tumor samples belonging to groups that have worse survival for LGG and SKCM cancer types. It was shown in^72^, that highly aneuploid cancer cells lose small chromosomes for cancer cell survival. Thus, from our analysis, we could suggest that cancers in PANoptosis High group of LGG and PANoptosis Low cluster of SKCM could preferentially lose small chromosomes to evade cell death^72^, and aneuploidy could play a contributing role in the worse survival prognosis of the patients in that cluster^19^.

By deconvoluting the bulk RNA-Seq^45^, we estimated significantly more M2 macrophages were present in the PANoptosis High cluster for LGG. The M2 macrophages are known to have tumor-promoting capabilities and can contribute to a worsened prognosis^73^. On average, significantly more M1 macrophages and cytotoxic CD8 + T-cells, along with enhanced activations of B-cells, Th1 cells, cytotoxic cells and NK CD56dim cells were seen, suggesting an active immune-infiltrated tumor microenvironment potentially driving better survival prognosis for the PANoptosis High cluster in SKCM^73^.

Finally, we correlated the PANoptosis score with the enrichment of oncogenic pathways and differentiated their activations between the two clusters, finding associations for LGG and SKCM cancers. The differential activation of immunosuppressive pathways such as angiogenesis, epithelial mesenchymal transition, TGF-beta signaling along with a loss in activity of anti-tumor immunity pathways such as NOS1 signature, MAPK up genes and telomerase extension by telomerase were multiple contributing factors for driving the difference between PANoptosis High and Low cluster, specifically in LGG. over, we identified that the proliferation pathway activations aligned with the differential prognostic association of PANoptosis for the three cancers. This suggests that tumors with high proliferative capacity tend to have worse survival prognosis in addition to the status of PANoptosis genes and thus should be an additional independent contributing factor for driving differential prognosis in these cancers (Supp. Fig. S7).

Additionally, we found that the hedgehog signaling pathway was activated in cancer cell lines with high PANoptosis scores (worse survival) for LGG and KIRC and low PANoptosis scores (worse survival) for SKCM. In breast cancer models, inhibiting the hedgehog signaling pathway reduces immune-suppressive innate and adaptive cells and enriches cytotoxic immune cells^75^. Thus, while the role of hedgehog signaling has been studied in the context of the immune microenvironment, its role in cancer cells conditioned by the PANoptosis phenotype requires further investigation.

Lastly, we observed that whenever the PANoptosis score was high, the barrier gene pathway had low activity in melanoma. This could be observed from the bulk RNA-Seq of SKCM cancers in TCGA, melanoma cancer cell lines in CCLE as well as in multiple cell types in a heterogenous melanoma single cell cohort. Lower activations of barrier genes suggest fewer mechanical barriers and a higher possibility of immune infiltration in the microenvironment^52^. The loss of activation of the barrier gene pathway, together with higher activation of the PANoptosis pathway, could drive better survival prognosis of patients with melanoma.

One limitation of these data is the difference in proteomics of tumors between the PANoptosis clusters owing to a lack of such data in TCGA. However, with the creation of The Clinical Proteomic Tumor Analysis Consoritum (CPTAC) such data are being collected for different cancer types including the availability of proteomic datasets for kidney cancer. Thus, the connection between the protein expression and PANoptosis phenotype might result in interesting findings and can be explored in a future follow-up work. Similarly, a comprehensive study focusing more on gaining insights at single-cell resolution can be undertaken as a future investigation. Finally, spatial transcriptional analysis^76^ could also help distinguish the tumor micro-environmental composition, activations, and cell–cell communications and directly determine the impact in differential prognosis of the PANoptosis clusters instead of using a bulk RNA-Seq deconvolution approach.

## Conclusion

Overall, our work builds upon the existing platform of using PANoptosis as a prognostic indicator and provides a comprehensive comparative analyses which identified the key contributing factors, including genomic alterations (aneuploidy), specific immune cell compositions (M2 macrophages), immune cell activations (activated cytotoxic CD8 + T-cells) and oncogenic pathway activations (proliferation and barrier gene pathway activations), that could be modulated to improve patient outcomes for at risk populations i.e., the PANoptosis High cluster for LGG and KIRC, and the PANoptosis Low cluster for SKCM.

### Supplementary Information

Supplementary Information.

## Data Availability

All code and relevant data are publicly available on Mendeley (https://doi.org/10.17632/7x237xf2m3.1).
