# Supplementary material for: Comparative analysis identifies genetic and molecular factors associated with prognostic clusters of PANoptosis in glioma, kidney and melanoma cancer
Source: Sci Rep. 2023 Nov 28;13:20962. doi: 10.1038/s41598-023-48098-1 (PMC10684528; doi:10.1038/s41598-023-48098-1)
Supplement: Supplementary file 1 — Supplementary Information. [file 41598_2023_48098_MOESM1_ESM.docx]

**
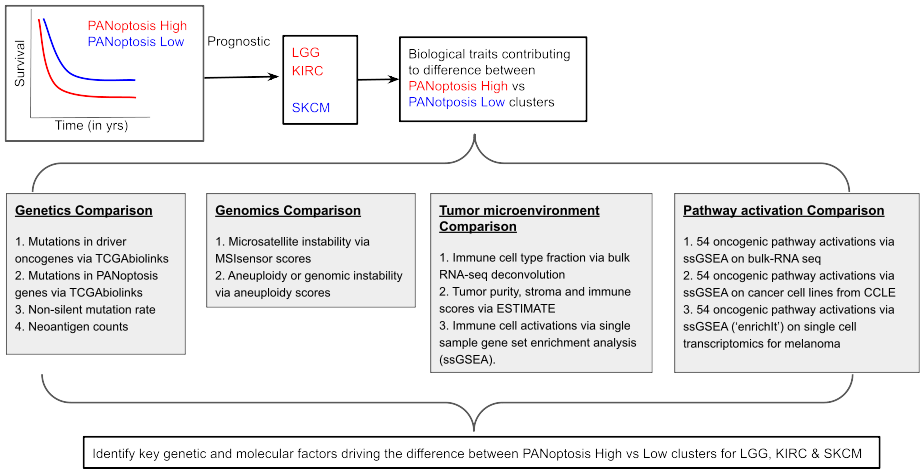
**

**Supplementary Figure 1: Overall study design.** A comprehensive workflow summarizing the overall study design is shown. LGG, Low Grade Glioma; KIRC, Kidney Renal Cell Carcinoma; SKCM, Skin Cutaneous Melanoma.

**Supplementary Figure 2**: **Comparison of somatic mutations in PANoptosis genes between PANoptosis High and PANoptosis Low clusters.**

**A-C)** Oncoplot comparing the difference in mutation profiles of PANoptosis genes between PANoptosis High and PANoptosis Low clusters for LGG (A), KIRC (B), and SKCM (C). Several of the PANoptosis genes were not mutated in the LGG and KIRC tumor samples and hence were not present in the oncoplot. Only a small number of PANoptosis genes had a high number of total somatic mutations in the PANoptosis High and Low clusters, thereby rendering the Chi-square test ineffective. Samples from the PANoptosis High group are depicted in red, and samples from the PANoptosis Low group are in blue.

**Supplementary Figure 3**: **A summary of the mutation profile for LGG driver oncogenes.**

**A-B)** A highlight of the total number of tumor samples with specific variant class, type, and single nucleotide variant (SNV) class along with the median number of variants per sample, variant classification summary per sample, and top 10 mutated driver oncogenes along with the percentage of tumor samples in which they were mutated for the **(A)** PANoptosis High and **(B)** PANoptosis Low clusters for LGG. Here, SNP refers to single nucleotide polymorphism; INS refers to insertion; and DEL refers to deletion.

**Supplementary Figure 4**: **A summary of the mutation profile for KIRC driver oncogenes.**

**A-B)** A highlight of the total number of tumor samples with specific variant class, type, and single nucleotide variant (SNV) class along with the median number of variants per sample, variant classification summary per sample, and top 10 mutated driver oncogenes along with the percentage of tumor samples in which they were mutated for the **(A)** PANoptosis High and **(B)** PANoptosis Low clusters for KIRC. Here, SNP refers to single nucleotide polymorphism; INS refers to insertion; and DEL refers to deletion.

**Supplementary Figure 5:** **A summary of the mutation profile of SKCM driver oncogenes.**

**A-B)** A highlight of the total number of tumor samples with specific variant class, type, and single nucleotide variant (SNV) class along with the median number of variants per sample, variant classification summary per sample, and top 10 mutated driver oncogenes along with the percentage of tumor samples in which they were mutated for the **(A)** PANoptosis High and **(B)** PANoptosis Low clusters for SKCM. Here, SNP refers to single nucleotide polymorphism; INS refers to insertion; and DEL refers to deletion.

**Supplementary Figure 6: Differential immune composition between PANoptosis High and PANoptosis Low clusters for LGG, KIRC, and SKCM.**

Comparison of the stromal, immune, and ESTIMATE (tumor purity) scores between the PANoptosis High and PANoptosis Low clusters of LGG, KIRC, and SKCM. These scores were calculated using the ESTIMATE (47) algorithm, and the statistical difference between them for PANoptosis High versus PANoptosis Low groups was obtained using the Wilcoxon rank-sum test with a **** *P*-value < 1 × 10^-5^ .

**Supplementary Figure 7: Pathway activity in PANoptosis High and Low clusters for LGG, KIRC, and SKCM.**

A heatmap highlighting the activity profiles of the 54 oncogenic pathways across the PANoptosis High and PANoptosis Low clusters from bulk RNA-Seq tumor samples estimated using the ssGSEA method for LGG, KIRC, and SKCM cancer types.


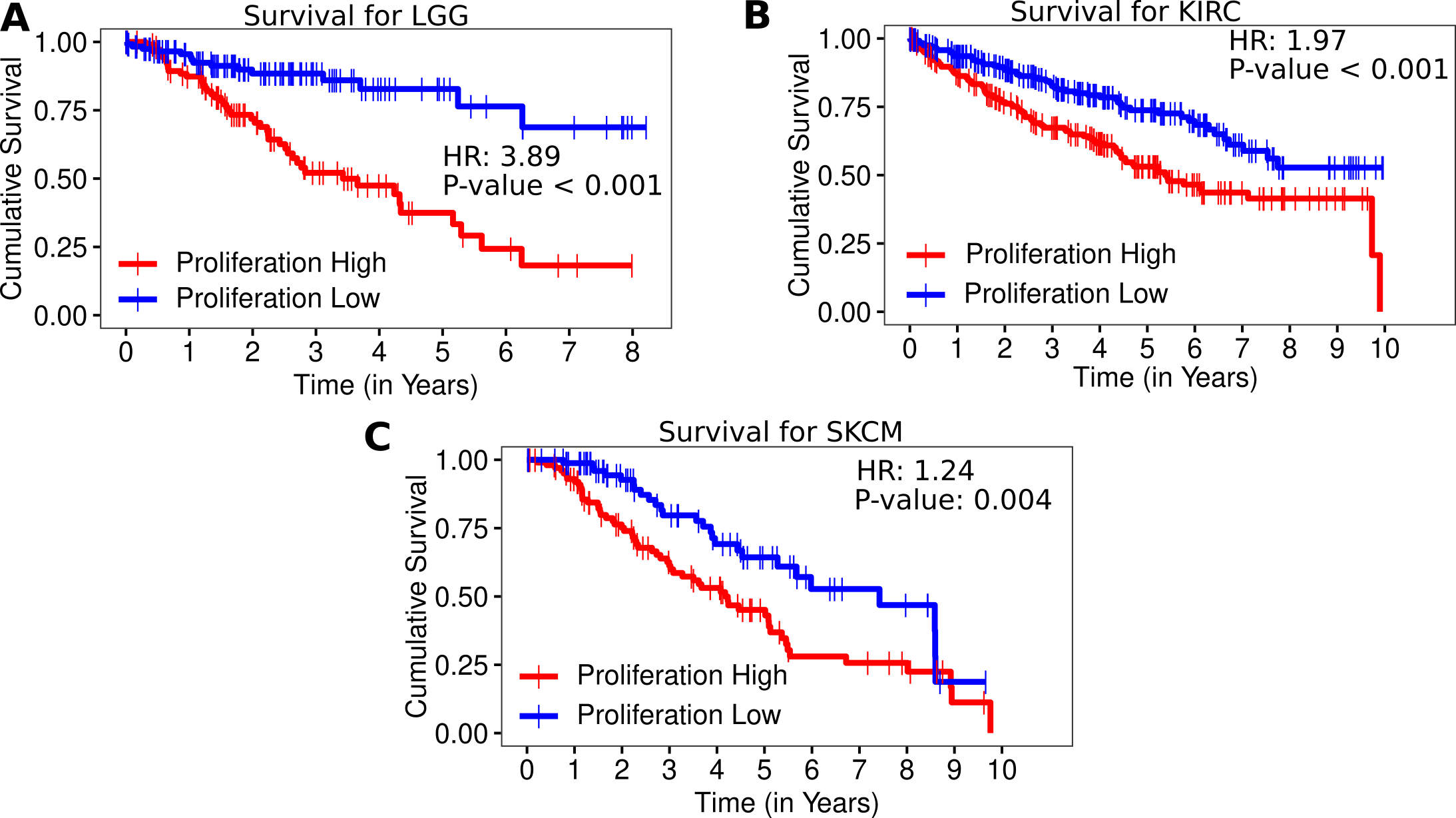


**Supplementary Figure 8**: **Prognostic association of proliferation pathway activation with overall survival in LGG, KIRC, and SKCM**.

**A-C)** Kaplan-Meier curves showing overall survival (OS) across the Proliferation High and Low groups in the three cancer types with significant differences in survival. The proliferation pathway activity was estimated using the single-sample gene set enrichment analysis (ssGSEA) method. The mean proliferation pathway activity was used for each cancer type to divide the patient samples into High and Low proliferation groups. Here, ‘HR’ corresponds to hazard Ratio.

**Supplementary Table 1: Mutation profiles in PANoptosis High vs Low clusters for LGG**​

**PANoptosis High vs PANoptosis Low**​

|  | | PANoptosis High | | | | | | | | | PANoptosis Low | | | | | | |
| --- | --- | --- | --- | --- | --- | --- | --- | --- | --- | --- | --- | --- | --- | --- | --- | --- | --- |
| ​ | ​ | **LOF**​ | **LOF**​ | **LOF**​ | **LOF**​ | **GOF**​ | **LOF**​ | **LOF**​ | **LOF**​ | ​ | **LOF**​ | **LOF**​ | **LOF**​ | **GOF**​ | **LOF**​ | **LOF**​ | ​ |
| ​ | Gene​ | Frame Shift Del​ | Frame Shift Ins​ | In Frame Del​ | In Frame Ins​ | Missense ​ | Nonsense​ | Splice Site​ | TSS​ | Mutated Samples​ | Frame Shift Del​ | Frame Shift Ins​ | In Frame Del​ | Missense​ | Nonsense​ | Splice Site​ | Mutated Samples​ |
| **HF**​ | *IDH1*​ | 0​ | 0​ | 0​ | 0​ | 50​ | 0​ | 0​ | 0​ | 50​ | 0​ | 0​ | 0​ | 123​ | 0​ | 0​ | 123​ |
| **HF**​ | *TP53*​ | 3​ | 2​ | 3​ | 0​ | 51​ | 4​ | 3​ | 0​ | 52​ | 0​ | 1​ | 1​ | 39​ | 0​ | 0​ | 35​ |
| **LOF**​ | *CIC*​ | 0​ | 0​ | 0​ | 0​ | 1​ | 1​ | 0​ | 0​ | 2​ | 23​ | 8​ | 2​ | 39​ | 4​ | 3​ | 65​ |
| **LOF**​ | *ATRX*​ | 14​ | 5​ | 1​ | 0​ | 12​ | 10​ | 2​ | 0​ | 39​ | 7​ | 5​ | 0​ | 2​ | 5​ | 4​ | 23​ |
| **HF**​ | *EGFR*​ | 0​ | 0​ | 0​ | 0​ | 26​ | 0​ | 0​ | 0​ | 20​ | 0​ | 0​ | 0​ | 3​ | 0​ | 0​ | 3​ |
| **HF**​ | *PIK3CA*​ | 0​ | 0​ | 0​ | 0​ | 12​ | 0​ | 0​ | 1​ | 13​ | 0​ | 0​ | 4​ | 12​ | 0​ | 0​ | 14​ |
| **LOF**​ | *NOTCH1*​ | 0​ | 0​ | 0​ | 0​ | 2​ | 0​ | 0​ | 0​ | 2​ | 1​ | 0​ | 12​ | 10​ | 1​ | 1​ | 22​ |
| **LOF**​ | *NF1*​ | 5​ | 3​ | 1​ | 0​ | 3​ | 5​ | 2​ | 0​ | 13​ | 2​ | 0​ | 0​ | 0​ | 0​ | 0​ | 2​ |
| **HF**​ | *IDH2*​ | 0​ | 0​ | 0​ | 1​ | 0​ | 0​ | 0​ | 0​ | 1​ | 0​ | 0​ | 0​ | 13​ | 0​ | 0​ | 13​ |
| **HF**​ | *PTEN*​ | 0​ | 2​ | 0​ | 0​ | 8​ | 2​ | 0​ | 1​ | 13​ | 0​ | 0​ | 0​ | 0​ | 0​ | 0​ | 0​ |
| **LOF**​ | *PIK3R1*​ | 0​ | 0​ | 0​ | 0​ | 2​ | 0​ | 1​ | 0​ | 3​ | 1​ | 0​ | 5​ | 2​ | 0​ | 0​ | 7​ |
| **HF**​ | *SMARCA4*​ | 0​ | 0​ | 1​ | 0​ | 1​ | 0​ | 0​ | 0​ | 2​ | 0​ | 0​ | 1​ | 7​ | 0​ | 0​ | 8​ |
| **LOF**​ | *ARID1A*​ | 1​ | 0​ | 0​ | 0​ | 0​ | 0​ | 0​ | 0​ | 1​ | 3​ | 0​ | 0​ | 1​ | 3​ | 0​ | 6​ |
| **LOF**​ | *BCOR*​ | 0​ | 1​ | 0​ | 0​ | 1​ | 0​ | 0​ | 0​ | 2​ | 3​ | 1​ | 0​ | 2​ | 0​ | 0​ | 6​ |
| **HF**​ | *FBN2*​ | 0​ | 0​ | 0​ | 0​ | 3​ | 0​ | 0​ | 0​ | 3​ | 1​ | 0​ | 0​ | 3​ | 1​ | 0​ | 5​ |
| **LOF**​ | *TCF12*​ | 1​ | 1​ | 0​ | 0​ | 0​ | 0​ | 0​ | 0​ | 2​ | 3​ | 1​ | 1​ | 0​ | 0​ | 1​ | 5​ |
| **HF**​ | *SETD2*​ | 0​ | 1​ | 0​ | 0​ | 3​ | 1​ | 0​ | 0​ | 4​ | 0​ | 0​ | 0​ | 1​ | 0​ | 0​ | 1​ |
| **HF**​ | *AHNAK2*​ | 0​ | 0​ | 0​ | 0​ | 2​ | 0​ | 0​ | 0​ | 2​ | 0​ | 0​ | 0​ | 3​ | 0​ | 0​ | 3​ |
| **HF**​ | *SYNE1*​ | 0​ | 0​ | 0​ | 0​ | 1​ | 0​ | 0​ | 0​ | 1​ | 0​ | 0​ | 0​ | 4​ | 0​ | 0​ | 4​ |

HF, hyper-functioning​

LOF, loss-of-function​

GF, gain-of-function

**Supplementary Table 2: Top Mutation profiles in PANoptosis High vs Low clusters for KIRC​**

**PANoptosis High vs PANoptosis Low**

|  | | PANoptosis High | | | | | | | | | | PANoptosis Low | | | | | | | |
| --- | --- | --- | --- | --- | --- | --- | --- | --- | --- | --- | --- | --- | --- | --- | --- | --- | --- | --- | --- |
| ​ | ​ | **LOFl**​ | **LOF**​ | **LOF**​ | **LOF**​ | **GOF**​ | **LOF**​ | **LOF**​ | **LOF**​ | **LOF**​ | ​ | **LOF**​ | **LOF**​ | **LOF**​ | **LOF**​ | **GOF**​ | **LOF**​ | **LOF**​ | ​ |
| ​ | Gene​ | Frame Shift Del​ | Frame Shift Ins​ | In Frame Del​ | In Frame Ins​ | Missense​ | Nonsense​ | Nonstop​ | Splice Site​ | TSS​ | Mutated Samples​ | Frame Shift Del​ | Frame Shift Ins​ | In Frame Del​ | In Frame Ins​ | Missense​ | Nonsense​ | Splice Site​ | Mutated Samples​ |
| **HF/LOF**​ | *VHL*​ | 17​ | 10​ | 0​ | 0​ | 34​ | 12​ | 1​ | 9​ | 0​ | 83​ | 16​ | 6​ | 3​ | 0​ | 27​ | 13​ | 5​ | 67​ |
| **HF**​ | *PBRM1*​ | 25​ | 8​ | 0​ | 0​ | 10​ | 18​ | 0​ | 10​ | 0​ | 68​ | 23​ | 2​ | 1​ | 1​ | 8​ | 16​ | 6​ | 57​ |
| **LOF**​ | *SETD2*​ | 9​ | 2​ | 0​ | 0​ | 7​ | 11​ | 0​ | 2​ | 0​ | 28​ | 4​ | 1​ | 0​ | 0​ | 3​ | 4​ | 3​ | 12​ |
| **LOF**​ | *BAP1*​ | 6​ | 0​ | 0​ | 0​ | 5​ | 7​ | 0​ | 4​ | 3​ | 24​ | 1​ | 1​ | 1​ | 0​ | 5​ | 2​ | 1​ | 10​ |
| **HF**​ | *MTOR*​ | 0​ | 0​ | 0​ | 0​ | 13​ | 0​ | 0​ | 0​ | 0​ | 12​ | 0​ | 0​ | 1​ | 0​ | 14​ | 0​ | 0​ | 14​ |
| **LOF**​ | *KDM5C*​ | 5​ | 0​ | 0​ | 1​ | 3​ | 5​ | 0​ | 0​ | 0​ | 13​ | 1​ | 0​ | 0​ | 0​ | 1​ | 1​ | 1​ | 4​ |
| **HF**​ | *ATM*​ | 4​ | 0​ | 0​ | 0​ | 3​ | 3​ | 0​ | 1​ | 0​ | 9​ | 1​ | 0​ | 0​ | 0​ | 4​ | 1​ | 0​ | 4​ |
| **HF**​ | *SPEN*​ | 2​ | 0​ | 0​ | 0​ | 9​ | 1​ | 0​ | 0​ | 0​ | 10​ | 0​ | 0​ | 0​ | 0​ | 5​ | 0​ | 0​ | 5​ |
| **HF**​ | *FBN2*​ | 1​ | 0​ | 0​ | 0​ | 6​ | 1​ | 0​ | 1​ | 0​ | 8​ | 0​ | 0​ | 0​ | 0​ | 6​ | 0​ | 0​ | 5​ |
| **LOF**​ | *PTEN*​ | 4​ | 1​ | 0​ | 0​ | 3​ | 2​ | 0​ | 2​ | 0​ | 11​ | 1​ | 0​ | 0​ | 0​ | 0​ | 1​ | 1​ | 2​ |
| **HF**​ | *AHNAK2*​ | 1​ | 0​ | 1​ | 0​ | 6​ | 0​ | 0​ | 0​ | 0​ | 8​ | 1​ | 0​ | 0​ | 0​ | 4​ | 1​ | 0​ | 6​ |
| **HF**​ | *SYNE1*​ | 1​ | 0​ | 0​ | 0​ | 6​ | 0​ | 0​ | 0​ | 0​ | 7​ | 0​ | 0​ | 0​ | 0​ | 4​ | 1​ | 0​ | 5​ |
| **HF**​ | *PRPF8*​ | 1​ | 0​ | 1​ | 0​ | 3​ | 1​ | 0​ | 0​ | 0​ | 6​ | 0​ | 0​ | 0​ | 0​ | 5​ | 0​ | 0​ | 5​ |
| **HF**​ | *TP53*​ | 0​ | 1​ | 0​ | 0​ | 5​ | 1​ | 0​ | 1​ | 0​ | 8​ | 0​ | 0​ | 0​ | 0​ | 1​ | 1​ | 1​ | 3​ |
| **HF**​ | *AKAP9*​ | 0​ | 0​ | 0​ | 0​ | 2​ | 1​ | 0​ | 0​ | 0​ | 3​ | 0​ | 0​ | 0​ | 0​ | 7​ | 0​ | 0​ | 6​ |
| **LOF**​ | *ARID1A*​ | 2​ | 1​ | 0​ | 0​ | 2​ | 0​ | 0​ | 0​ | 0​ | 5​ | 2​ | 0​ | 0​ | 0​ | 1​ | 2​ | 0​ | 5​ |
| **HF**​ | *NEB*​ | 2​ | 0​ | 0​ | 0​ | 5​ | 0​ | 0​ | 0​ | 0​ | 7​ | 1​ | 1​ | 0​ | 0​ | 1​ | 0​ | 0​ | 3​ |
| **HF**​ | *RANBP2*​ | 0​ | 0​ | 0​ | 0​ | 6​ | 0​ | 0​ | 0​ | 0​ | 3​ | 0​ | 0​ | 0​ | 0​ | 3​ | 0​ | 0​ | 3​ |
| **HF**​ | *ATRX*​ | 0​ | 0​ | 0​ | 0​ | 5​ | 0​ | 0​ | 0​ | 0​ | 5​ | 1​ | 0​ | 0​ | 0​ | 2​ | 0​ | 0​ | 3​ |
| **HF**​ | *EP300*​ | 0​ | 0​ | 0​ | 0​ | 4​ | 1​ | 0​ | 0​ | 0​ | 5​ | 1​ | 0​ | 0​ | 0​ | 2​ | 0​ | 0​ | 3​ |
| **HF**​ | *MAP3K4*​ | 0​ | 0​ | 0​ | 0​ | 3​ | 0​ | 0​ | 0​ | 0​ | 3​ | 0​ | 0​ | 0​ | 0​ | 4​ | 1​ | 0​ | 4​ |
| **HF**​ | *MED13*​ | 1​ | 0​ | 1​ | 0​ | 3​ | 1​ | 0​ | 0​ | 0​ | 6​ | 1​ | 0​ | 0​ | 0​ | 1​ | 0​ | 0​ | 2​ |
| **HF**​ | *NF1*​ | 0​ | 0​ | 0​ | 0​ | 2​ | 1​ | 0​ | 0​ | 0​ | 3​ | 2​ | 0​ | 0​ | 0​ | 2​ | 1​ | 0​ | 4​ |
| **HF**​ | *SMARCA4*​ | 0​ | 0​ | 0​ | 0​ | 4​ | 0​ | 0​ | 1​ | 0​ | 5​ | 0​ | 0​ | 0​ | 0​ | 2​ | 0​ | 1​ | 3​ |
| **HF**​ | *BRCA2*​ | 0​ | 0​ | 0​ | 0​ | 3​ | 0​ | 0​ | 0​ | 0​ | 3​ | 0​ | 0​ | 0​ | 0​ | 4​ | 0​ | 0​ | 4​ |
| **HF**​ | *MLLT4*​ | 0​ | 0​ | 0​ | 0​ | 2​ | 0​ | 0​ | 0​ | 0​ | 2​ | 0​ | 0​ | 0​ | 0​ | 3​ | 1​ | 1​ | 5​ |
| **HF**​ | *NAV3*​ | 0​ | 0​ | 0​ | 0​ | 3​ | 0​ | 0​ | 0​ | 0​ | 3​ | 0​ | 0​ | 0​ | 0​ | 3​ | 0​ | 1​ | 3​ |
| **HF**​ | *PLEC*​ | 0​ | 0​ | 0​ | 0​ | 4​ | 1​ | 0​ | 0​ | 0​ | 5​ | 0​ | 0​ | 0​ | 0​ | 2​ | 0​ | 0​ | 2​ |
| **HF**​ | *ABCB1*​ | 0​ | 0​ | 0​ | 0​ | 4​ | 0​ | 0​ | 0​ | 0​ | 3​ | 0​ | 0​ | 0​ | 0​ | 1​ | 1​ | 0​ | 2​ |
| **LOF**​ | *CDK12*​ | 1​ | 0​ | 0​ | 1​ | 1​ | 1​ | 0​ | 0​ | 0​ | 4​ | 0​ | 0​ | 0​ | 0​ | 1​ | 0​ | 1​ | 2​ |
| **HF**​ | *CHD4*​ | 0​ | 0​ | 0​ | 0​ | 1​ | 0​ | 0​ | 0​ | 0​ | 1​ | 0​ | 0​ | 0​ | 0​ | 5​ | 0​ | 0​ | 5​ |
| **HF**​ | *CLTC*​ | 1​ | 0​ | 0​ | 0​ | 2​ | 0​ | 0​ | 0​ | 0​ | 3​ | 0​ | 1​ | 0​ | 0​ | 1​ | 0​ | 1​ | 3​ |
| **HF**​ | *HUWE1*​ | 0​ | 0​ | 0​ | 0​ | 3​ | 0​ | 0​ | 0​ | 0​ | 3​ | 0​ | 0​ | 0​ | 0​ | 3​ | 0​ | 0​ | 3​ |
| **HF**​ | *LRRK2*​ | 1​ | 0​ | 0​ | 0​ | 2​ | 0​ | 0​ | 0​ | 0​ | 3​ | 0​ | 0​ | 0​ | 0​ | 3​ | 0​ | 0​ | 3​ |
| **HF**​ | *NCOR2*​ | 0​ | 0​ | 0​ | 0​ | 3​ | 0​ | 0​ | 0​ | 0​ | 3​ | 0​ | 1​ | 0​ | 0​ | 2​ | 0​ | 0​ | 3​ |
| **HF**​ | *NSD1*​ | 0​ | 0​ | 0​ | 0​ | 3​ | 0​ | 0​ | 1​ | 0​ | 4​ | 1​ | 0​ | 0​ | 0​ | 1​ | 0​ | 0​ | 2​ |
| **HF**​ | *SMC3*​ | 0​ | 0​ | 0​ | 0​ | 0​ | 0​ | 0​ | 0​ | 0​ | 0​ | 0​ | 0​ | 0​ | 0​ | 6​ | 0​ | 0​ | 5​ |
| **HF**​ | *SOS1*​ | 0​ | 0​ | 0​ | 0​ | 3​ | 0​ | 0​ | 0​ | 0​ | 3​ | 1​ | 0​ | 0​ | 0​ | 2​ | 0​ | 0​ | 3​ |
| **LOF**​ | *STAG2*​ | 1​ | 0​ | 0​ | 0​ | 1​ | 2​ | 0​ | 0​ | 0​ | 4​ | 1​ | 0​ | 0​ | 0​ | 1​ | 0​ | 0​ | 2​ |
| **HF**​ | *TSC2*​ | 0​ | 0​ | 0​ | 0​ | 1​ | 0​ | 0​ | 1​ | 0​ | 2​ | 0​ | 0​ | 0​ | 0​ | 3​ | 0​ | 1​ | 3​ |
| **HF**​ | *APC*​ | 1​ | 0​ | 0​ | 0​ | 2​ | 0​ | 0​ | 0​ | 0​ | 3​ | 0​ | 1​ | 0​ | 0​ | 1​ | 0​ | 0​ | 2​ |
| **LOF**​ | *ARHGAP35*​ | 1​ | 0​ | 1​ | 0​ | 0​ | 1​ | 0​ | 0​ | 0​ | 3​ | 0​ | 0​ | 0​ | 0​ | 2​ | 0​ | 0​ | 2​ |
| **HF**​ | *ASXL1*​ | 0​ | 0​ | 0​ | 0​ | 4​ | 1​ | 0​ | 0​ | 0​ | 5​ | 0​ | 0​ | 0​ | 0​ | 0​ | 0​ | 0​ | 0​ |
| **HF**​ | *BRCA1*​ | 0​ | 0​ | 0​ | 0​ | 2​ | 0​ | 0​ | 0​ | 0​ | 2​ | 0​ | 0​ | 0​ | 0​ | 3​ | 0​ | 0​ | 2​ |
| **HF**​ | *DMD*​ | 0​ | 0​ | 0​ | 0​ | 3​ | 0​ | 0​ | 1​ | 0​ | 4​ | 0​ | 0​ | 0​ | 0​ | 1​ | 0​ | 0​ | 1​ |
| **HF**​ | *DNMT3A*​ | 1​ | 0​ | 0​ | 0​ | 2​ | 0​ | 0​ | 0​ | 0​ | 3​ | 1​ | 0​ | 0​ | 0​ | 1​ | 0​ | 0​ | 2​ |
| **HF**​ | *DOCK2*​ | 0​ | 0​ | 0​ | 0​ | 3​ | 0​ | 0​ | 0​ | 0​ | 3​ | 0​ | 0​ | 0​ | 0​ | 2​ | 0​ | 0​ | 2​ |
| **HF**​ | *HSPA8*​ | 0​ | 0​ | 0​ | 0​ | 2​ | 0​ | 0​ | 0​ | 0​ | 2​ | 0​ | 0​ | 0​ | 0​ | 2​ | 1​ | 0​ | 2​ |
| **LOF**​ | *MAX*​ | 2​ | 0​ | 0​ | 0​ | 0​ | 0​ | 0​ | 0​ | 0​ | 2​ | 1​ | 0​ | 0​ | 0​ | 1​ | 0​ | 1​ | 3​ |
| **HF**​ | *NCOR1*​ | 0​ | 0​ | 0​ | 0​ | 3​ | 0​ | 0​ | 0​ | 0​ | 3​ | 0​ | 0​ | 0​ | 0​ | 2​ | 0​ | 0​ | 2​ |
| **HF**​ | *NUP98*​ | 0​ | 0​ | 0​ | 0​ | 3​ | 0​ | 0​ | 0​ | 0​ | 2​ | 0​ | 0​ | 0​ | 0​ | 2​ | 0​ | 0​ | 2​ |
| **HF**​ | *PDGFRA*​ | 0​ | 0​ | 0​ | 0​ | 1​ | 0​ | 0​ | 0​ | 0​ | 1​ | 0​ | 0​ | 0​ | 0​ | 3​ | 1​ | 0​ | 3​ |
| **HF**​ | *PIK3CA*​ | 0​ | 0​ | 0​ | 0​ | 4​ | 0​ | 0​ | 0​ | 0​ | 4​ | 0​ | 0​ | 0​ | 0​ | 0​ | 1​ | 0​ | 1​ |
| **HF**​ | *RNF43*​ | 0​ | 0​ | 0​ | 0​ | 4​ | 0​ | 0​ | 0​ | 0​ | 4​ | 0​ | 0​ | 0​ | 0​ | 1​ | 0​ | 0​ | 1​ |
| **HF**​ | *TAF1*​ | 0​ | 0​ | 0​ | 0​ | 4​ | 0​ | 0​ | 0​ | 0​ | 4​ | 0​ | 0​ | 0​ | 0​ | 1​ | 0​ | 0​ | 1​ |
| **HF**​ | *ZFHX3*​ | 1​ | 0​ | 0​ | 0​ | 0​ | 1​ | 0​ | 0​ | 0​ | 2​ | 0​ | 0​ | 0​ | 0​ | 3​ | 0​ | 0​ | 3​ |
| **HF**​ | *ZMYM2*​ | 0​ | 0​ | 0​ | 0​ | 3​ | 0​ | 0​ | 0​ | 0​ | 3​ | 0​ | 0​ | 0​ | 0​ | 2​ | 0​ | 0​ | 2​ |

HF, hyper-functioning​

LOF, loss-of-function​

GF, gain-of-function

**Supplementary Table 3: Top50  Mutation profiles in PANoptosis High vs Low clusters for SKCM**​**​**

**PANoptosis High vs PANoptosis Low**

|  | | PANoptosis High | | | | | | | | | PANoptosis Low | | | | | | |
| --- | --- | --- | --- | --- | --- | --- | --- | --- | --- | --- | --- | --- | --- | --- | --- | --- | --- |
| ​ | ​ | **LOF**​ | **LOF**​ | **LOF**​ | **LOF**​ | **GOF**​ | **LOF**​ | **LOF**​ | **LOF**​ | ​ | **LOF**​ | **LOF**​ | **LOF**​ | **GOF**​ | **LOF**​ | **LOF**​ | ​ |
| ​ | Gene​ | Frame Shift Del​ | Frame Shift Ins​ | In Frame Del​ | In Frame Ins​ | Missense​ | Nonsense​ | Splice Site​ | TSS​ | Mutated Samples​ | Frame Shift Del​ | Frame Shift Ins​ | In Frame Del​ | Missense​ | Nonsense​ | Splice Site​ | Mutated Samples​ |
| **HF**​ | *SYNE1*​ | 0​ | 0​ | 0​ | 0​ | 86​ | 13​ | 2​ | 0​ | 53​ | 0​ | 0​ | 0​ | 18​ | 4​ | 1​ | 13​ |
| **HF**​ | *BRAF*​ | 0​ | 0​ | 0​ | 0​ | 91​ | 0​ | 0​ | 0​ | 77​ | 0​ | 0​ | 0​ | 14​ | 0​ | 0​ | 14​ |
| **HF**​ | *NEB*​ | 0​ | 0​ | 0​ | 0​ | 73​ | 8​ | 0​ | 0​ | 47​ | 0​ | 0​ | 0​ | 17​ | 2​ | 0​ | 11​ |
| **HF**​ | *KIAA1109*​ | 0​ | 0​ | 0​ | 0​ | 38​ | 3​ | 2​ | 0​ | 30​ | 0​ | 0​ | 0​ | 15​ | 1​ | 1​ | 8​ |
| **HF**​ | *NRAS*​ | 0​ | 0​ | 0​ | 0​ | 39​ | 0​ | 1​ | 0​ | 38​ | 0​ | 0​ | 0​ | 18​ | 0​ | 1​ | 19​ |
| **HF**​ | *AHNAK2*​ | 0​ | 0​ | 0​ | 0​ | 43​ | 1​ | 0​ | 0​ | 30​ | 0​ | 0​ | 0​ | 11​ | 3​ | 0​ | 10​ |
| **HF**​ | *ARID2*​ | 1​ | 0​ | 1​ | 0​ | 21​ | 18​ | 0​ | 0​ | 27​ | 1​ | 0​ | 0​ | 10​ | 3​ | 1​ | 10​ |
| **HF**​ | *FBN2*​ | 0​ | 1​ | 0​ | 0​ | 34​ | 5​ | 1​ | 0​ | 29​ | 0​ | 0​ | 0​ | 11​ | 0​ | 0​ | 8​ |
| **HF**​ | *MECOM*​ | 0​ | 0​ | 0​ | 0​ | 32​ | 2​ | 0​ | 0​ | 28​ | 0​ | 0​ | 0​ | 18​ | 0​ | 0​ | 13​ |
| **LOF**​ | *NF1*​ | 1​ | 0​ | 0​ | 0​ | 18​ | 16​ | 3​ | 0​ | 27​ | 3​ | 1​ | 0​ | 3​ | 6​ | 1​ | 11​ |
| **HF**​ | *KALRN*​ | 0​ | 0​ | 0​ | 0​ | 36​ | 5​ | 1​ | 0​ | 28​ | 0​ | 0​ | 0​ | 7​ | 0​ | 1​ | 5​ |
| **HF**​ | *BCLAF1*​ | 0​ | 0​ | 0​ | 0​ | 34​ | 1​ | 0​ | 0​ | 30​ | 0​ | 0​ | 0​ | 12​ | 0​ | 0​ | 7​ |
| **HF**​ | *CFH*​ | 0​ | 1​ | 0​ | 0​ | 29​ | 3​ | 0​ | 0​ | 20​ | 0​ | 0​ | 0​ | 8​ | 1​ | 0​ | 7​ |
| **HF**​ | *ASPM*​ | 0​ | 0​ | 0​ | 0​ | 34​ | 2​ | 0​ | 0​ | 24​ | 0​ | 0​ | 0​ | 3​ | 2​ | 0​ | 5​ |
| **HF**​ | *DMD*​ | 0​ | 0​ | 0​ | 0​ | 28​ | 2​ | 0​ | 0​ | 26​ | 0​ | 0​ | 0​ | 8​ | 2​ | 0​ | 6​ |
| **HF**​ | *HUWE1*​ | 0​ | 0​ | 0​ | 0​ | 31​ | 1​ | 0​ | 0​ | 28​ | 0​ | 0​ | 0​ | 6​ | 1​ | 0​ | 6​ |
| **HF**​ | *RNF213*​ | 0​ | 0​ | 0​ | 0​ | 28​ | 7​ | 0​ | 0​ | 25​ | 0​ | 0​ | 0​ | 4​ | 0​ | 0​ | 3​ |
| **HF**​ | *AKAP9*​ | 0​ | 0​ | 0​ | 0​ | 29​ | 4​ | 1​ | 0​ | 26​ | 0​ | 0​ | 0​ | 3​ | 0​ | 0​ | 2​ |
| **HF**​ | *CHD8*​ | 0​ | 0​ | 0​ | 0​ | 22​ | 4​ | 2​ | 0​ | 24​ | 0​ | 0​ | 0​ | 6​ | 1​ | 1​ | 6​ |
| **HF**​ | *NAV3*​ | 0​ | 0​ | 0​ | 0​ | 21​ | 2​ | 3​ | 0​ | 19​ | 0​ | 0​ | 0​ | 9​ | 0​ | 0​ | 5​ |
| **HF**​ | *TP53*​ | 2​ | 0​ | 0​ | 0​ | 15​ | 9​ | 2​ | 0​ | 25​ | 0​ | 0​ | 1​ | 4​ | 0​ | 1​ | 6​ |
| **HF**​ | *ABCB1*​ | 1​ | 0​ | 0​ | 0​ | 23​ | 2​ | 0​ | 0​ | 21​ | 0​ | 0​ | 0​ | 7​ | 0​ | 0​ | 5​ |
| **HF**​ | *APC*​ | 0​ | 0​ | 0​ | 0​ | 21​ | 2​ | 0​ | 0​ | 20​ | 2​ | 0​ | 0​ | 7​ | 0​ | 0​ | 7​ |
| **HF**​ | *LIFR*​ | 0​ | 0​ | 0​ | 0​ | 19​ | 2​ | 0​ | 0​ | 18​ | 0​ | 0​ | 0​ | 9​ | 1​ | 0​ | 4​ |
| **HF**​ | *SPEN*​ | 0​ | 0​ | 0​ | 0​ | 22​ | 2​ | 1​ | 0​ | 19​ | 0​ | 0​ | 0​ | 5​ | 0​ | 0​ | 3​ |
| **HF**​ | *PLEC*​ | 0​ | 0​ | 0​ | 0​ | 19​ | 1​ | 1​ | 0​ | 19​ | 0​ | 0​ | 0​ | 7​ | 1​ | 0​ | 6​ |
| **HF**​ | *LRRK2*​ | 0​ | 0​ | 0​ | 0​ | 20​ | 2​ | 0​ | 0​ | 19​ | 0​ | 0​ | 0​ | 4​ | 0​ | 1​ | 4​ |
| **HF**​ | *PRPF8*​ | 0​ | 0​ | 0​ | 0​ | 15​ | 3​ | 2​ | 0​ | 16​ | 0​ | 0​ | 0​ | 7​ | 0​ | 0​ | 5​ |
| **HF**​ | *ARHGAP32*​ | 0​ | 0​ | 0​ | 0​ | 21​ | 1​ | 0​ | 0​ | 19​ | 0​ | 0​ | 0​ | 4​ | 0​ | 0​ | 4​ |
| **HF**​ | *FN1*​ | 0​ | 0​ | 0​ | 0​ | 18​ | 2​ | 2​ | 0​ | 17​ | 0​ | 0​ | 0​ | 3​ | 1​ | 0​ | 4​ |
| **HF**​ | *LEPR*​ | 2​ | 0​ | 0​ | 0​ | 14​ | 3​ | 2​ | 0​ | 18​ | 0​ | 0​ | 0​ | 5​ | 0​ | 0​ | 4​ |
| **HF**​ | *SMARCA4*​ | 0​ | 0​ | 0​ | 0​ | 21​ | 0​ | 0​ | 0​ | 19​ | 0​ | 0​ | 0​ | 5​ | 0​ | 0​ | 4​ |
| **HF**​ | *ZFHX3*​ | 0​ | 0​ | 0​ | 0​ | 18​ | 2​ | 0​ | 0​ | 17​ | 0​ | 0​ | 0​ | 6​ | 0​ | 0​ | 6​ |
| **HF**​ | *CAD*​ | 0​ | 0​ | 0​ | 0​ | 18​ | 1​ | 0​ | 0​ | 16​ | 0​ | 0​ | 0​ | 6​ | 0​ | 0​ | 5​ |
| **HF**​ | *DICER1*​ | 0​ | 0​ | 0​ | 0​ | 14​ | 4​ | 0​ | 0​ | 16​ | 0​ | 0​ | 0​ | 6​ | 0​ | 1​ | 7​ |
| **HF**​ | *FGFR2*​ | 0​ | 0​ | 0​ | 0​ | 18​ | 0​ | 0​ | 1​ | 19​ | 0​ | 0​ | 0​ | 6​ | 0​ | 0​ | 5​ |
| **HF**​ | *NCOR2*​ | 0​ | 0​ | 0​ | 0​ | 18​ | 0​ | 0​ | 0​ | 16​ | 0​ | 0​ | 0​ | 7​ | 0​ | 0​ | 7​ |
| **HF**​ | *NSD1*​ | 0​ | 0​ | 0​ | 0​ | 19​ | 2​ | 0​ | 0​ | 19​ | 0​ | 0​ | 0​ | 3​ | 0​ | 1​ | 4​ |
| **HF**​ | *ATM*​ | 0​ | 0​ | 0​ | 0​ | 14​ | 2​ | 3​ | 0​ | 16​ | 0​ | 0​ | 0​ | 4​ | 1​ | 0​ | 5​ |
| **HF**​ | *ATR*​ | 0​ | 0​ | 0​ | 0​ | 21​ | 1​ | 0​ | 0​ | 17​ | 0​ | 0​ | 0​ | 1​ | 0​ | 1​ | 2​ |
| **HF**​ | *CDKN2A*​ | 2​ | 0​ | 0​ | 0​ | 10​ | 8​ | 0​ | 0​ | 19​ | 0​ | 0​ | 1​ | 1​ | 1​ | 1​ | 4​ |
| **HF**​ | *MTOR*​ | 0​ | 0​ | 0​ | 0​ | 16​ | 1​ | 0​ | 0​ | 15​ | 0​ | 0​ | 0​ | 5​ | 0​ | 2​ | 6​ |
| **HF**​ | *NLRP3*​ | 0​ | 0​ | 0​ | 0​ | 17​ | 1​ | 2​ | 0​ | 18​ | 0​ | 0​ | 0​ | 4​ | 0​ | 0​ | 4​ |
| **HF**​ | *TAF1*​ | 0​ | 0​ | 0​ | 0​ | 17​ | 1​ | 2​ | 0​ | 15​ | 0​ | 0​ | 0​ | 4​ | 0​ | 0​ | 4​ |
| **HF**​ | *TP53BP1*​ | 0​ | 0​ | 0​ | 0​ | 22​ | 1​ | 0​ | 0​ | 19​ | 0​ | 0​ | 0​ | 1​ | 0​ | 0​ | 1​ |
| **HF**​ | *DOCK2*​ | 0​ | 0​ | 0​ | 0​ | 17​ | 1​ | 0​ | 0​ | 15​ | 0​ | 0​ | 0​ | 5​ | 0​ | 0​ | 5​ |
| **HF**​ | *FLT3*​ | 0​ | 0​ | 0​ | 0​ | 15​ | 1​ | 1​ | 0​ | 15​ | 0​ | 0​ | 0​ | 6​ | 0​ | 0​ | 4​ |
| **HF**​ | *NCOR1*​ | 0​ | 0​ | 0​ | 0​ | 12​ | 2​ | 1​ | 0​ | 14​ | 0​ | 0​ | 0​ | 8​ | 0​ | 0​ | 8​ |

HF, hyper-functioning​

LOF, loss-of-function​

GF, gain-of-function
